# Engineering active sites on hierarchical transition bimetal oxides/sulfides heterostructure array enabling robust overall water splitting

**DOI:** 10.1038/s41467-020-19214-w

**Published:** 2020-10-29

**Authors:** Panlong Zhai, Yanxue Zhang, Yunzhen Wu, Junfeng Gao, Bo Zhang, Shuyan Cao, Yanting Zhang, Zhuwei Li, Licheng Sun, Jungang Hou

**Affiliations:** 1 State Key Laboratory of Fine Chemicals, School of Chemical Engineering, Dalian University of Technology, 116024 Dalian, P. R. China; 2 Laboratory of Materials Modification by Laser, Ion and Electron Beams, Dalian University of Technology, Ministry of Education, 116024 Dalian, P. R. China; 3 College of Science, Westlake University, 310024 Hangzhou, P. R. China; 4 Department of Chemistry, School of Engineering Sciences in Chemistry, Biotechnology and Health, KTH Royal Institute of Technology, 10044 Stockholm, Sweden

**Keywords:** Electrocatalysis, Electrocatalysis, Nanoscale materials

## Abstract

Rational design of the catalysts is impressive for sustainable energy conversion. However, there is a grand challenge to engineer active sites at the interface. Herein, hierarchical transition bimetal oxides/sulfides heterostructure arrays interacting two-dimensional MoO_x_/MoS_2_ nanosheets attached to one-dimensional NiO_x_/Ni_3_S_2_ nanorods were fabricated by oxidation/hydrogenation-induced surface reconfiguration strategy. The NiMoO_x_/NiMoS heterostructure array exhibits the overpotentials of 38 mV for hydrogen evolution and 186 mV for oxygen evolution at 10 mA cm^−2^, even surviving at a large current density of 500 mA cm^−2^ with long-term stability. Due to optimized adsorption energies and accelerated water splitting kinetics by theory calculations, the assembled two-electrode cell delivers the industrially relevant current densities of 500 and 1000 mA cm^−2^ at record low cell voltages of 1.60 and 1.66 V with excellent durability. This research provides a promising avenue to enhance the electrocatalytic performance of the catalysts by engineering interfacial active sites toward large-scale water splitting.

## Abstract (web summary)

While water splitting is an appealing carbon-neutral strategy for renewable energy generation, there is a need to develop new active, cost-effective catalysts. Here, authors prepare a nickel-molybdenum oxide/sulfide heterojunctions as bifunctional H_2_ and O_2_ evolution electrocatalysts.

## Introduction

The generation of clean energy from water electrolysis is a feasible solution to overcome the problems of energy issues^1^. The sustainable alternative for hydrogen generation is electrocatalytic water splitting, involving hydrogen evolution reaction (HER) and oxygen evolution reaction (OER)^2^. Generally, noble materials, Pt for HER and RuO_2_ or IrO_2_ for OER, are typical electrocatalysts. Nevertheless, the practical application is limited by the use of noble materials owing to the scarcity and the high cost. To this end, it is interesting to produce bifunctional materials by the integration of OER and HER catalysts towards water splitting in various media^3^. To address the challenges, catalyzing HER, OER and overall water splitting have been conducted by extensive catalysts, such as oxides, hydroxides, phosphides, nitrides and chalcogenides^4–11^. Thus, it is urgently needed to design earth-abundant and low-cost non-noble-metal catalysts for industrial applications.

Among various materials, Mo- and Ni-based sulfides are promising transition-metal electrocatalysts. To improve the performance of these catalysts, various strategies, such as morphology engineering, defect engineering, and heterostructure engineering have been adopted in this field. Architectural nanostructures have been controlled by the synthesis regulation of the electrocatalysts owe to inherent anisotropy and high flexibility^5–8^. Inspired by the advantages of the architectures, the integration of different nanostructures can effectively optimize the electrocatalytic performance. For example, MoO_3_ nanodots supported on MoS_2_ monolayer, MoNi_4_ anchored MoO_2_ cuboids or MoO_3-x_ nanorods and NiS_2_/N-NiMoO_4_ nanosheets/nanowires have been produced for the excellent electrocatalytic water splitting^12–15^, providing an appealing platform with the hierarchical nanostructures. Apart from morphology engineering, the hybrids can be extensively constructed by use of different transition-metal electrocatalysts through heterostructure engineering, regulating electron transfer and active site as well as the activity owe to the construction of coupling interfaces and the synergistic effect of the heterostructures. For instance, a large number of the heterostructures, such as NiMo/NiMoO_*x*_^8^, Co_3_O_4_/Fe_0.33_Co_0.66_P^16^, Ni_2_P/NiP_2_^17^, NiFe(OH)_*x*_/FeS^18^, Pt_2_W/WO_3_^19^, CuCo/CuCoO_*x*_^20^, Co(OH)_2_/PANI^21^, FeOOH/Co/FeOOH^22^, Co_0.85_Se/NiFe/graphene^23^, Ni_3_N/VN^24^, NiCu–NiCuN^25^, have been extensively synthesized for the enhanced electrochemical activities. Typically, sulfides-based heterostructures, such as CoS-doped β-Co(OH)_2_/MoS_2+*x*_^26^, MoS_2_/Fe_5_Ni_4_S_8_^27^, MoS_2_/Ni_3_S_2_^28^, NiS_2_/MoS_2_^29^, MoS_2_/Co_9_S_8_/Ni_3_S_2_/Ni^30^, and MoS_2_/(Co,Fe,Ni)_9_S_8_ coupled FeCoNi-based arrays^31^, have been systematically explored for the improved activities of electrochemical water splitting. With regard to transition-metal dichalcogenides, MoS_2_ and Ni_3_S_2_ materials have been substantially explored as HER electrocatalysts^32–35^. However, the HER performance of transition metal sulfides is limited by poor charge transport, low active site reactivity, and inefficient electrical contact with the supported catalysts^36^. Especially, the generation of S–H_ads_ bonds (H atoms adsorption, H_ads_) on the surface of metal sulfides is beneficial for H adsorption, while it is difficult to conduct the conversion of the H_ads_ to H_2_^36,37^. However, the OER performance of metal sulfides remains far from satisfactory^27–31^. Owe to long-time durability as major obstacle, there is less report about the electrocatalysts, delivering large catalytic current densities (e.g., 500 and 1000 mA cm^−2^) for practical application^38–41^. Based on the above-mentioned analysis, it is essential to design the rational heterostructures through the combined regulation of architectural morphology and heterostructures, engineering active sites, optimizing energy adsorption, and accelerating water splitting kinetics towards large-scale electrolysis.

Herein, three-dimensional (3D) NiMoO_*x*_/NiMoS heterostructure array is fabricated by surface reconfiguration strategy through oxygen plasma as oxidation treatment and subsequent hydrogenation regulation by use of NiMoS architecture as the precursor, interacting two-dimensional (2D) MoO_*x*_/MoS_2_ nanosheets attached to one-dimensional (1D) NiO_*x*_/Ni_3_S_2_ nanorods array. As-synthesized NiMoO_*x*_/NiMoS array presents the remarkable electrocatalytic performance, achieving the low overpotentials of 38, 89, 174, and 236 mV for HER and 186, 225, 278, and 334 mV for OER at 10, 100, 500, and 1000 mA cm^−2^, even surviving at large current densities of 100 and 500 mA cm^−2^ with long-term stability. The remarkable electrocatalytic performance of transition bimetal oxides/sulfides heterostructure array as the industrially promising electrocatalyst is ascribed to not only the simultaneous modulation of component and geometric structure, but also the systematic optimization of charge transfer, abundant electrocatalytic active sites, and exceptionally synergistic effect of the heterostructure interfaces. The turnover frequency (TOF) of NiMoO_*x*_/NiMoS array at the overpotential of 100 mV is ~45 times higher than that of NiMoS array. Density functional theory calculations reveal that the coupling interface between NiMoO_*x*_ and NiMoS optimizes adsorption energies and accelerates water splitting kinetics, thus promoting the electrocatalytic performance. Especially, the assembled two-electrode cell by use of NiMoO_*x*_/NiMoS array delivers the industrially required current densities of 500 and 1000 mA cm^−2^ at the low cell voltages of 1.60 and 1.66 V, along with excellent durability, thus holding great promise for industrial water splitting application.

## Results

### Synthesis and characterization

**Fig. 1:**
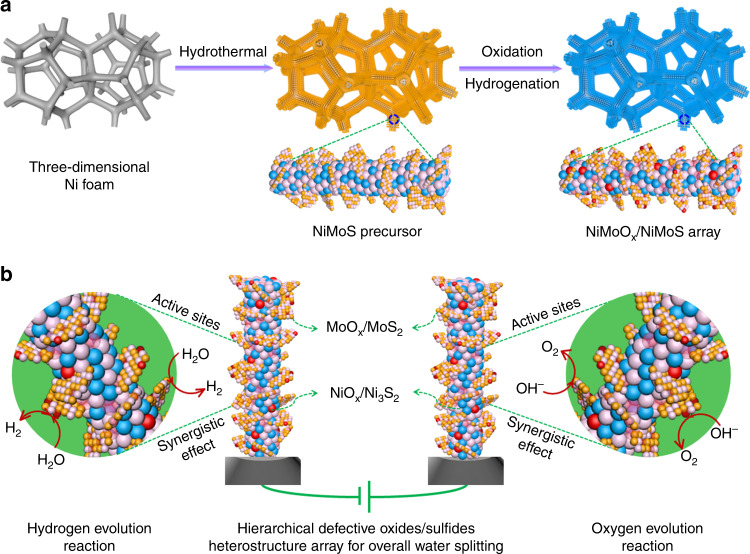
Schematic representation of synthesis and overall water splitting. **a** Synthesis illustration of transition bimetal oxides/sulfides heterostructure array. **b** NiMoO_*x*_/NiMoS array as two-electrode-cell towards large-scale electrolysis. Colored balls represent various elements (blue: Mo, pink: S, red: O, yellow: Ni).

The hierarchical NiMoO_*x*_/NiMoS array was fabricated by oxidation/hydrogenation-induced surface reconfiguration strategy by use of NiMoS precursor, assembling as two-electrode cell towards industrially electrocatalytic water splitting (Fig. 1 and Supplementary Fig. 1). To determine the crystal structure, X-ray diffraction (XRD) patterns of NiMoS-based arrays are showed (Supplementary Fig. 2). Based on the hydrothermal reaction, the representative peaks of the precursors can be assigned to the planes of MoS_2_ phase (JCPDS No. 37-1492) and Ni_3_S_2_ phase (JCPDS No. 44-1418), confirming the formation of individual MoS_2_ and Ni_3_S_2_ as well as MoS_2_/Ni_3_S_2_ heterostructure as the precursors (Supplementary Fig. 2a). After oxygen plasma as oxidation treatment and subsequent hydrogenation regulation, several MoO_3_ (JCPDS No.47-1320), MoO_2_ (JCPDS No. 50-0739), and NiO (JCPDS No.44-1159) phases as well as the mixed MoO_3_/MoO_2_/NiO/Ni phases are observed in MoS_2_, Ni_3_S_2_, and MoS_2_/Ni_3_S_2_ (Supplementary Fig. 2b). Thus, all above-mentioned results demonstrate the successful formation of NiO_x_/Ni_3_S_2_, MoO_*x*_/MoS_2_, and NiMoO_*x*_/NiMoS heterostructure arrays.

**Fig. 2:**
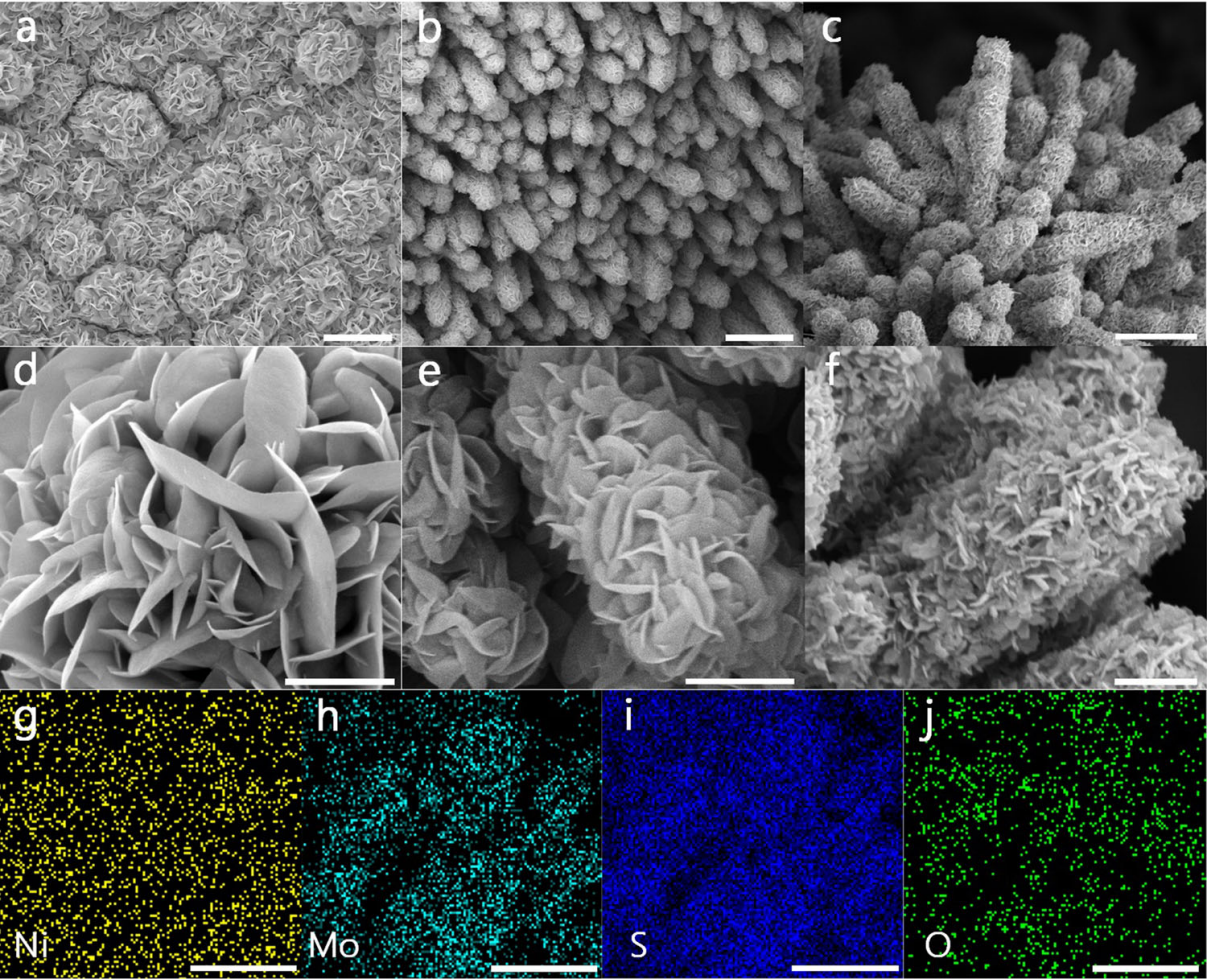
Morphological and structural characterizations. SEM images of **a**, **d** MoS_2_, **b**, **e** NiMoS, **c**, **f** NiMoO_*x*_/NiMoS. **g**–**j** Elemental mapping images of NiMoO_*x*_/NiMoS. Scale bar, **a**–**c** 5 μm; **d**–**f** 1 μm; **g**–**j** 10 μm.

To confirm the geometric morphologies of individual arrays by scanning electron microscope (SEM), as shown in Fig. 2a, d, MoS_2_ nanosheets as the precursor with an average size over 1 μm are homogeneously supported on the conductive substrate. While the rough surface of Ni_3_S_2_ array as the precursor is observed (Supplementary Fig. 3). Interestingly, two-dimensional (2D) MoS_2_ nanosheets with an average size below 1 μm are attached to one-dimensional (1D) Ni_3_S_2_ nanorods array on 3D foam substrate, resulting into the formation of hierarchical MoS_2_/Ni_3_S_2_ (denoted as NiMoS) heterostructure array (Fig. 2b, e). After the oxidation/hydrogenation treatment of NiMoS array, there is no obvious change upon the main morphology for 3D NiMoO_*x*_/NiMoS heterostructure array. However, the small size and rough surface of MoS_2_ nanosheets in NiMoO_*x*_/NiMoS array are observed in comparison of MoS_2_ in NiMoS array (Fig. 2cf). Meanwhile, the energy-dispersive X-ray (EDX) spectra and elemental mapping (Fig. 2 and Supplementary Fig. 4) indicate the molar content of MoO_*x*_/MoS_2_ about 6.1% and the homogeneous element distribution in NiMoO_*x*_/NiMoS array. Thus, the above-mentioned analysis indicates the formation of NiMoO_*x*_/NiMoS array as 3D integrated architectures.

**Fig. 3:**
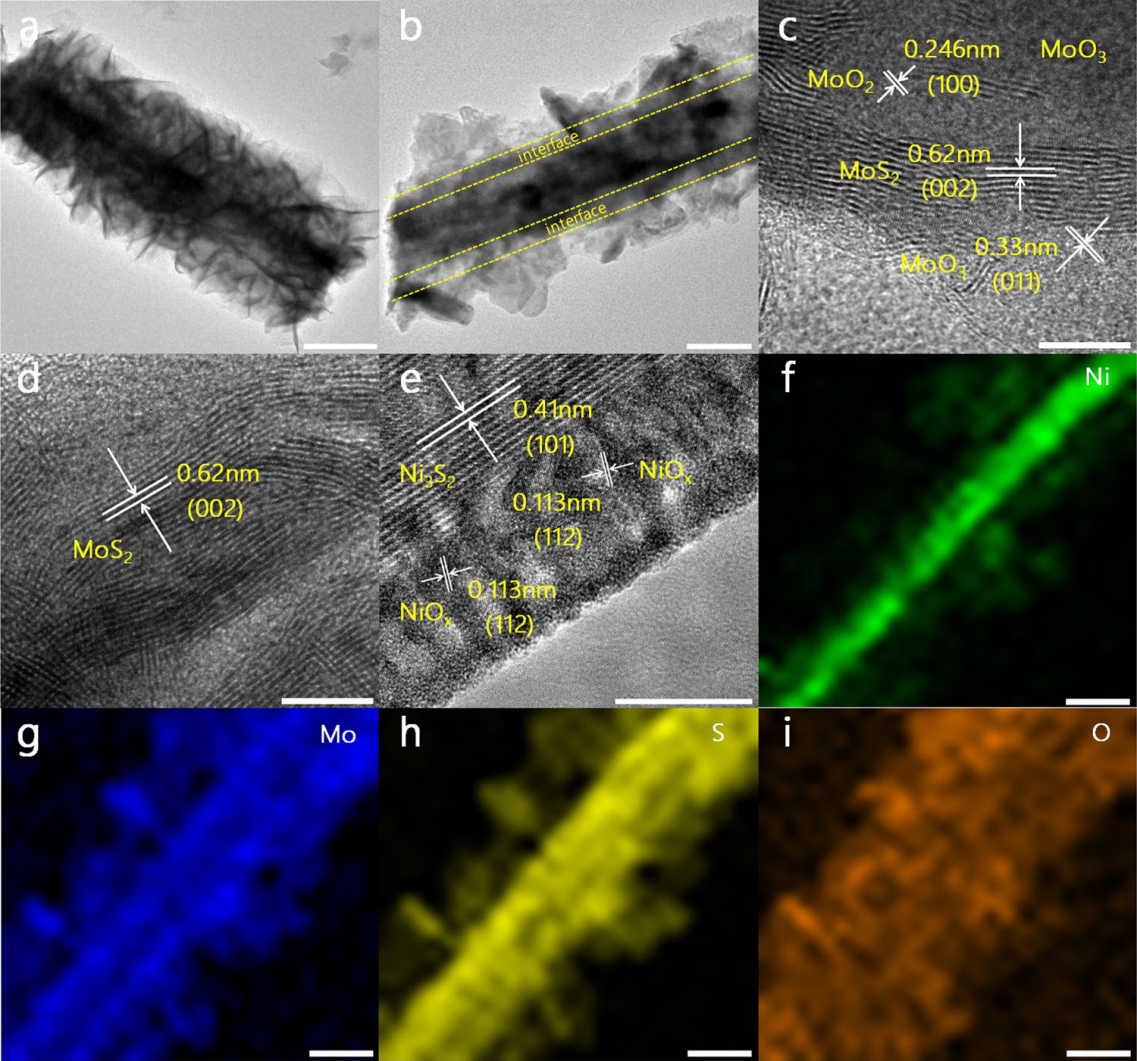
Morphological and structural characterizations. TEM and HRTEM images of **a**, **d** NiMoS and **b**, **c**, **e** NiMoO_x_/NiMoS. **f**–**i** Elemental distribution mapping of Ni, Mo, S, and O in NiMoO_*x*_/NiMoS. Scale bar, **a**, **b** 500 nm; **c**–**e** 5 nm; **f–i** 200 nm.

To check the details of the morphology, transition electron microscope (TEM) and high-resolution TEM observations verify the architectures of NiMoS and NiMoO_*x*_/NiMoS heterostructure arrays, indicating that MoS_2_ and MoO_*x*_/MoS_2_ nanosheets are attached to Ni_3_S_2_ and NiO_*x*_/Ni_3_S_2_ nanorods arrays, respectively (Fig. 3). Compared to MoS_2_ and Ni_3_S_2_ in NiMoS nanostructures, the characteristic lattice fringes of 0.62, 0.33, and 0.246 nm can be assigned to the (002) plane of MoS_2_, (011) plane of MoO_3,_ and (100) plane of MoO_2_ and even more, the (101) plane of Ni_3_S_2_ and the (112) plane of NiO_*x*_ can be proven by the lattice fringes of 0.41 and 0.113 nm in NiMoO_*x*_/NiMoS heterostructures. Typically, the arrangements of MoO_x_ and NiO_*x*_ layers are observed on the surface of MoS_2_ and Ni_3_S_2_, indicating the formation of NiMoO_*x*_/NiMoS heterostructure array. Moreover, the elemental mappings by high-angle annular dark-field scanning transmission electron microscopy (HAADF-STEM) confirm the uniform distribution of Ni, Mo, S, and O (Fig. 3 and Supplementary Fig. 5). Therefore, the whole results of SEM and TEM analysis confirm the formation of 3D NiMoO_x_/NiMoS heterostructure array as the integrated architectures.

**Fig. 4:**
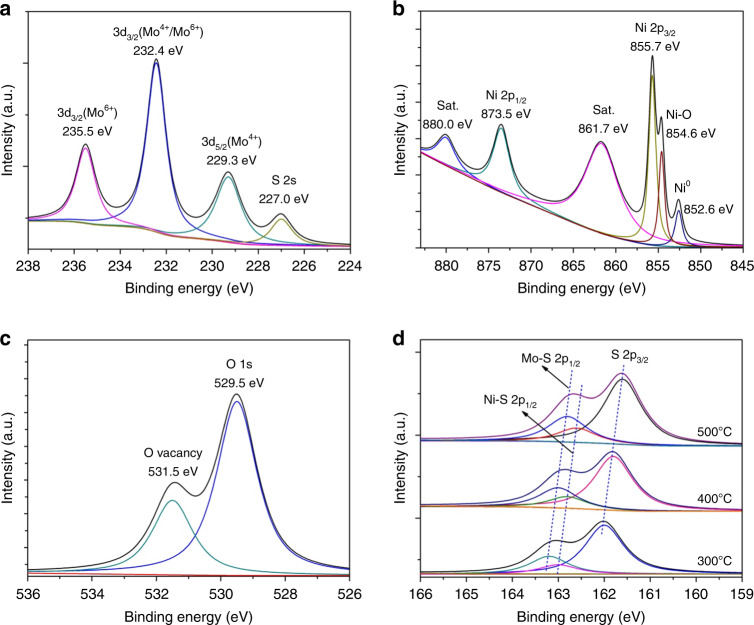
XPS spectra of NiMoO_x_/NiMoS. High-resolution XPS signals of **a** Mo 3*d*, **b** Ni 2*p*, **c** O 1s, **d** S 2*p* of NiMoO_*x*_/NiMoS array with different thermal treatment temperatures.

To conduct the chemical valences of the heterostructures, X-ray photoelectron spectroscopy (XPS) spectrum has been tested in Fig. 4. With regard to Mo 3*d* regions, the main peak could be split into two distinct peaks of Mo 3*d*_5/2_ (229.1 eV) and Mo 3*d*_3/2_ (232.4 eV), indicating the dominance of Mo^4+^ in NiMoS (Supplementary Fig. 6)^8,42^. The peaks at 855.2, 861.5, 872.9 and 879.5 eV can be indexed to Ni 2*p*_3/2_ and Ni 2*p*_1/2_ orbitals as well as two satellites in NiMoS (Supplementary Fig. 6)^25^. However, the signals at 229.3, 232.4, and 235.5 eV can be indexed to Mo^4+^ 3*d*_5/2_, Mo^4+/6+^ 3*d*_3/2,_ and Mo^6+^ 3*d*_3/2_ orbitals, confirming the existence of Mo^4+^ and Mo^6+^ in NiMoO_*x*_/NiMoS owe to the formation of MoO_x_^26^. For Ni 2p orbitals, there is a shift upon the peak positions and the two new peaks at 854.6 and 852.6 eV, demonstrating the existence of Ni–O bonds and metallic Ni^0^ and the formation of NiO_*x*_ species in NiMoO_*x*_/NiMoS^8,25^. Typically, the signals at 529.5 and 531.5 eV for O 1s belong to typical metal-oxygen bonds and oxygen vacancies in NiMoO_*x*_/NiMoS heterostructure^8^. With regard to S 2*p* peaks, the negative shift is observed in NiMoO_x_/NiMoS with the increasing temperature of thermal treatment, demonstrating the loss of S and the formation of S vacancies^43^. The similar phenomenon of O 1*s* and S 2*p* is observed in MoO_*x*_/MoS_2_ and NiO_*x*_/Ni_3_S_2_ heterostructures (Supplementary Fig. 7–8). Thus, the combined analysis demonstrates the successful synthesis of hierarchical transition bimetal oxides/sulfides heterostructure array.

### Electrocatalytic HER performance

**Fig. 5:**
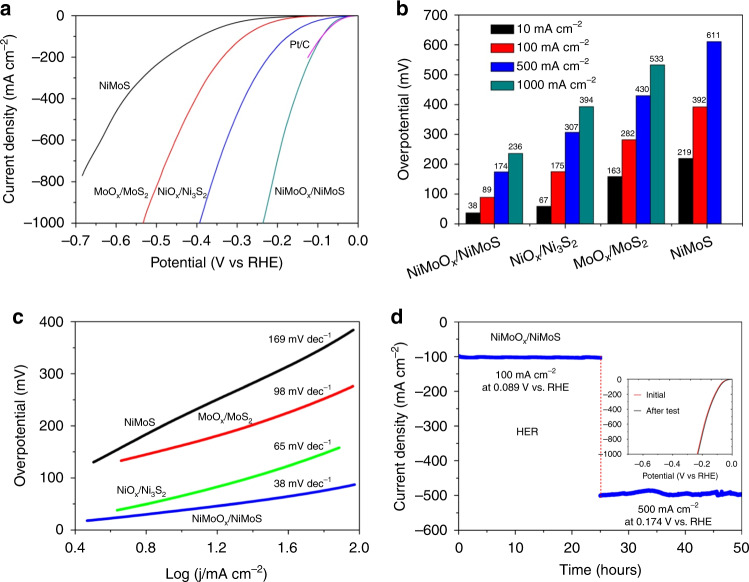
HER catalytic performance. **a** HER polarization curves, **b** overpotentials at typical current densities, **c** Tafel slopes of NiMoS, MoO_*x*_/MoS_2_, NiO_*x*_/Ni_3_S_2,_ and NiMoO_x_/NiMoS. **d** Time-dependent current density curves of NiMoO_*x*_/NiMoS at typical potentials. Inset: polarization curves of NiMoO_*x*_/NiMoS for the stability test.

The electrocatalytic performance of various arrays in the three-electrode system was conducted through a linear scan voltammogram (LSV) in 1 M KOH solution at 25 °C. The polarization curves of NiMoO_*x*_/NiMoS, MoO_*x*_/MoS_2_, NiO_*x*_/Ni_3_S_2,_ and NiMoS heterostructure arrays are presented in Fig. 5a, together with commercial Pt/C and Ni foam (Supplementary Fig. 9). In comparison of NiMoS (219, 392, and 611 mV), MoO_*x*_/MoS_2_ (163, 282, and 430 mV), NiO_*x*_/Ni_3_S_2_ (67, 175, and 307 mV), NiMoO_*x*_/NiMoS array delivers the current densities of 10, 100, and 500 mA cm^−2^ at the low overpotentials of 38, 89 and 174 mV, respectively, even requiring a low overpotential of 236 mV at a large current density of 1000 mA cm^−2^ towards HER (Fig. 5b). It is worth mentioning that NiMoO_*x*_/NiMoS array could surpass commercial Pt/C catalyst at the high overpotentials while comparable HER activity at the low potentials. Compared to most reported HER catalysts (Supplementary Table 1), the overpotential of NiMoO_*x*_/NiMoS array at a current density of 10 mA cm^−2^ is smaller than those of MoS_2_ (170 mV)^44^, CoFeZr oxides (104 mV)^6^, CoS-Co(OH)_2_@MoS_2+*x*_ (140 mV)^26^, MoS_2_/Fe_5_Ni_4_S_8_ (120 mV)^27^, MoS_2_/Ni_3_S_2_ (110 mV)^28^, MoS_2_/Co_9_S_8_/Ni_3_S_2_ (113 mV)^30^, and O-CoMoS (97 mV)^42^, etc. To regulate the capacities of charge transfer and active sites of NiMoS, it is interesting to determine the precise condition of plasma oxidation and hydrogenation treatment (Supplementary Fig. 10), indicating the best oxygen plasma power of 100 W and appropriate hydrogenation temperature of 400 °C of NiMoO_*x*_/NiMoS array. To conduct HER kinetic mechanism, the lowest Tafel slope (Fig. 5c), 38 mV per decade of NiMoO_*x*_/NiMoS array is obtained in comparison of NiO_*x*_/Ni_3_S_2_ (65 mV dec^−1^), MoO_*x*_/MoS_2_ (98 mV dec^−1^), NiMoS (169 mV dec^−1^), indicating the rapid HER kinetics of NiMoO_x_/NiMoS array owe to the advantages of the construction of 3D heterostructured architectures and the introduction of the defects. After the analysis of electrochemical impedance spectroscopy (EIS), the lowest charge transfer resistance of NiMoO_*x*_/NiMoS array due to the generation of defective species and metallic Ni is obtained in comparison of NiO_*x*_/Ni_3_S_2_, MoO_x_/MoS_2,_ and NiMoS (Supplementary Fig. 10). To explore the intrinsic electrocatalytic performance of each active sites, the turnover frequency (TOF) is calculated (Supplementary Table 2–3). The TOF value of NiMoO_*x*_/NiMoS array (1.97 s^−1^) at the overpotential of 100 mV is ~45 times higher than that of NiMoS array (0.0435 s^−1^). Moreover, mass activity, 436 A g^−1^ of NiMoO_*x*_/NiMoS array is calculated at the overpotential of 200 mV (Supplementary Fig. 11), which is better than other non-nobel metal electrocatalysts (Supplementary Table 4). Generally, the electrochemically active surface area (ECSA) is regarded as an estimation of active sites and is proportional to the double-layer capacitance (*C*_dl_)^45–47^. The highest *C*_dl_ values of NiMoO_*x*_/NiMoS array among all catalysts implies the maximum electroactive area (Supplementary Fig. 12). Moreover, the current of NiMoO_*x*_/NiMoS and commercial Pt/C supported on Ni plate was normalized to ECSA (Supplementary Figs. 13–16), demonstrating a higher instrinsic activity of NiMoO_x_/NiMoS catalyst in comparison of commercial Pt/C. Owe to the stability as pivot criterion for practical application, the time-dependent current density curves confirm that there is no obvious change upon the current densities of 100 and 500 mA cm^−2^ at 0.089 and 0.174 V vs. RHE over 50 h (Fig. 5d). Afterwards, the amount of hydrogen evolution of NiMoO_x_/NiMoS array is measured in comparison of theoretical quantity (Supplementary Fig. 17), presenting a promising Faradaic efficiency of 99.6 ± 0.3% towards real water splitting into hydrogen. Based on the above-mentioned analysis, the synergistic action of morphology and heterostructure engineering upon NiMoO_x_/NiMoS array can modulate the unique architectures, optimize the charge transfer and catalytic active sites, and thus improve HER performance.

### Electrocatalytic OER performance

**Fig. 6:**
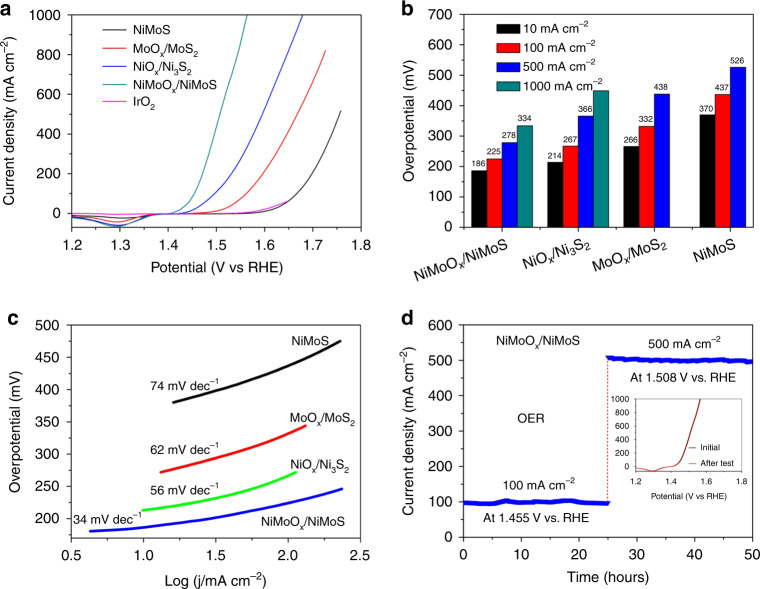
OER catalytic performance. **a** OER polarization curves, **b** overpotentials at typical current densities, **c** Tafel slopes of NiMoS, MoO_*x*_/MoS_2_, NiO_*x*_/Ni_3_S_2_, and NiMoO_*x*_/NiMoS. **d** Time-dependent current density curves of NiMoO_*x*_/NiMoS at typical potentials. Inset: polarization curves of NiMoO_*x*_/NiMoS for the durability test.

In general, the efficiency is always limited by OER as major barrier for overall water splitting. In our system, NiMoO_*x*_/NiMoS array exhibits the best OER performance among all arrays, together with commercial IrO_2_ catalyst and Ni foam (Fig. 6a and Supplementary Fig. 18). In comparison of NiMoS (370, 437, and 526 mV), MoO_*x*_/MoS_2_ (266, 332, and 438 mV), NiO_*x*_/Ni_3_S_2_ (214, 267, and 366 mV), as-synthesized NiMoO_x_/NiMoS array presents the low overpotentials of 186, 225, and 278 mV at current densities of 10, 100, and 500 mA cm^−2^, and delivers a large current density of 1000 mA cm^−2^ at 334 mV towards OER (Fig. 6b), satisfying the requirements for commercial electrocatalytic application (for example, *j* ≥ 500 mA cm^−2^ at *η* ≤ 300 mV)^48–51^. Compared to most reported OER catalysts (Supplementary Table 5), the overpotential of NiMoO_*x*_/NiMoS array at 10 mA cm^−2^ is still lower than those of O-CoMoS (272 mV)^42^, CoS-Co(OH)_2_@MoS_2+*x*_ (380 mV)^26^, MoS_2_/Fe_5_Ni_4_S_8_ (204 mV)^27^, MoS_2_/Ni_3_S_2_ (218 mV)^28^, and iron-substrate-derived electrocatalyst (269 mV)^48^, etc. Especially, the influence of oxygen plasma power and hydrogenation temperature upon the OER performance of NiMoO_*x*_/NiMoS array is determined (Supplementary Fig. 19), confirming the best plasma power of 100 W and thermal treatment temperature at 400 °C of NiMoO_*x*_/NiMoS array. To in-depth understand the OER kinetic mechanism, the lowest Tafel slope, 34 mV per decade of NiMoO_*x*_/NiMoS is achieved in comparison of NiO_*x*_/Ni_3_S_2_ (56 mV dec^−1^), MoO_*x*_/MoS_2_ (62 mV dec^−1^), NiMoS (74 mV dec^−1^), demonstrating the fast OER kinetics of NiMoO_x_/NiMoS (Fig. 6c). Remarkably, the largest *C*_dl_ value of 21.5 mF cm^–2^ of NiMoO_x_/NiMoS is obtained by the evaluation of ECSA among all arrays (Supplementary Fig. 20), indicating the production of abundant active sites in NiMoO_*x*_/NiMoS array. Especially, the high ECSA of NiMoO_*x*_/NiMoS array confirms the advantages of the exposured component and geometric structures of sufficient electrocatalytic active sites. Interestingly, the high-valence Mo and Ni species are obtained in NiMoO_*x*_/NiMoS array during the OER process (Supplementary Fig. 24), indicating the possible generation of hydroxyl oxides as the actual surface active sites and thus enhancing the OER activities owe to the synergistic action of 3D architectures and the heterostructures. In particular, NiMoO_*x*_/NiMoS array can preserve OER activities at 100 and 500 mA cm^−2^ with the potentials of 1.455 and 1.508 V vs. RHE over 50 h (Fig. 6d), indicating the fascinating OER stability. Typically, the amount of oxygen evolution of NiMoO_x_/NiMoS array is measured in comparison of theoretical quantity (Supplementary Fig. 21), presenting OER Faradaic efficiency of 97.5 ± 0.4% owe to the synergistic effect of the morphology and heterostructure engineering.

### Electrocatalytic performance for overall water splitting

**Fig. 7:**
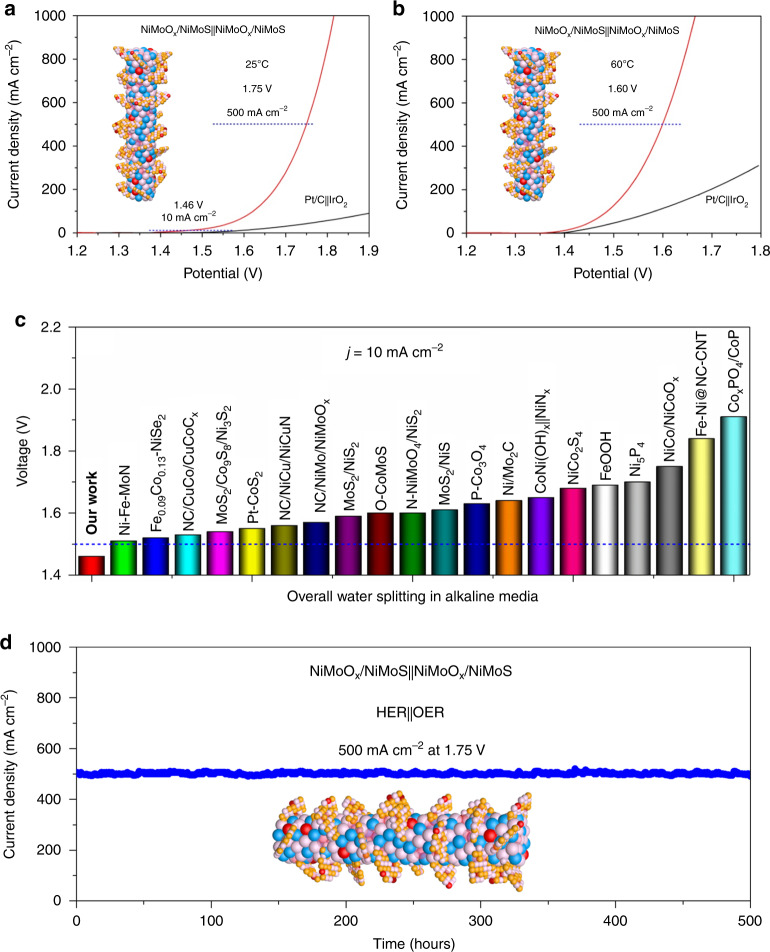
Electrocatalytic performance for overall water splitting. **a**, **b** Polarization curves by two-electrode system in **a** 1 M KOH at 25 °C and **b** 6 M KOH at 60 °C. **c** Comparison of the cell voltage at 10 mA cm^−2^ for NiMoO_*x*_/NiMoS with previously reported catalysts^8,15,20,25,30,42,47,52–64^. **d** Chronoamperometric test at 1.75 V in 1.0 M KOH at 25 °C.

Inspired by excellent HER and OER performance, NiMoO_*x*_/NiMoS array was assembled as cathode and anode in the two-electrode system. Impressively, the robust catalytic performance is achieved by as-synthesized NiMoO_*x*_/NiMoS||NiMoO_*x*_/NiMoS electrode (Fig. 7a), requiring the low cell voltages of 1.46, 1.62, 1.75, and 1.82 V at 10, 100, 500, and 1000 mA cm^−2^ in 1 M KOH at 25 °C. In comparison of Ni–Fe–MoN^52^, Fe_0.09_Co_0.13_-NiSe_2_^53^, NC/CoCu/CoCuO_x_^20^, MoS_2_/Co_9_S_8_/Ni_3_S_2_^30^, Pt-CoS_2_^47^, NC/NiCu/NiCuN^25^, NC/NiMo/NiMoO_*x*_^8^, MoS_2_/NiS_2_^54^, O-CoMoS^42^, N-NiMoO_4_/NiS_2_^15^, MoS_2_/NiS^55^, P-Co_3_O_4_^56^, Ni/Mo_2_C^57^, CoNi(OH)_x_ | NiN_*x*_^58^, NiCo_2_S_4_^59^, FeOOH^60^, Ni_5_P_4_^61^, NiCo/NiCoO_*x*_^62^, Fe-Ni@NC-CNT^63^, Co_*x*_PO_4_/CoP^64^, and commercial Pt/C||IrO_2_ electrodes (Fig. 7c and Supplementary Table 6), the lower voltage at 10 mA cm^−2^ is obtained for NiMoO_*x*_/NiMoS array. Owe to excellent electrocatalytic performance, the two-electrode cell can also be evaluated by a 1.5 V AAA battery (Supplementary Fig. 22). Based on the analysis of the superaerophobicity by bubble contact tests (Supplementary Fig. 23), the superior bubble contact angle, 151.2° of NiMoO_*x*_/NiMoS is obtained, demonstrating that this typical architecture could facilitate the release of the evolved gas bubbles and thus avoid the block of the catalyst active site. To be interesting, the hydrogen and oxygen bubbles escape effectively from the surface of NiMoO_*x*_/NiMoS array (Supplementary Movie). Moreover, the industrial environment is employed to explore the potential for industrialization applications. Typically, the record low voltages of 1.60 and 1.66 V of the two-electrode system in 6 M KOH solution at 60 °C are achieved for the industrial current densities of 500 and 1000 mA cm^−2^, respectively, and it is still better than that of Pt/C||IrO_2_ couple (Fig. 7b and Supplementary Tabel 7). Compared to the reported electrocatalysts with the large current densities (e.g., 500 and 1000 mA cm^−2^), such as NiMoN@NiFeN^65^, nickel-cobalt complexes hybridized MoS_2_^66^, Ni-P-B/paper^49^, NiVIr-LDH ||NiVRu-LDH^50^, phosphorus-doped Fe_3_O_4_^51^, graphdiyne-sandwiched layered double-hydroxide nanosheets^67^, N,S-coordinated Ir nanoclusters embedded on N,S-doped graphene^68^, Co_3_Mo/Cu^69^, and FeP/Ni_2_P hybrid^70^, all aforementioned analysis confirm that as-prepared NiMoO_*x*_/NiMoS array could be served as promising industrial candidate for overall water splitting. With regard to the operating stability as important metric, this typical two-electrode cell can maintain the excellent electrocatalytic activity at a large current density of 500 mA cm^−2^ at the voltage of 1.75 V over 500 h without obvious degradation in 1 M KOH solution at 25 °C (Fig. 7d). After HER, there is no obvious change upon the binding energies of various metal ions (Supplementary Fig. 24). However, the positive shift of two peaks located at 856.3 and 874.1 eV is observed in the XPS of Ni 2p, demonstrating that the oxidation of Ni^2+^ to high valence state of Ni^3+^, alone with the existence of new peak at 869.05 eV (Supplementary Fig. 24), thus indicating the formation of hydroxides and oxyhydroxides as the real active sites during OER process^30–34^. Although the hydroxides and oxyhydroxides are formed on the surface of NiMoO_x_/NiMoS array, there is no apparent change upon the morphology of the heterostructures (Supplementary Fig. 25), indicating the superior stability. Based on the above analysis, it is proven that NiMoO_*x*_/NiMoS array is excellent and stable system for overall water splitting, presenting the industrial hope.

### First-principles calculations

**Fig. 8:**
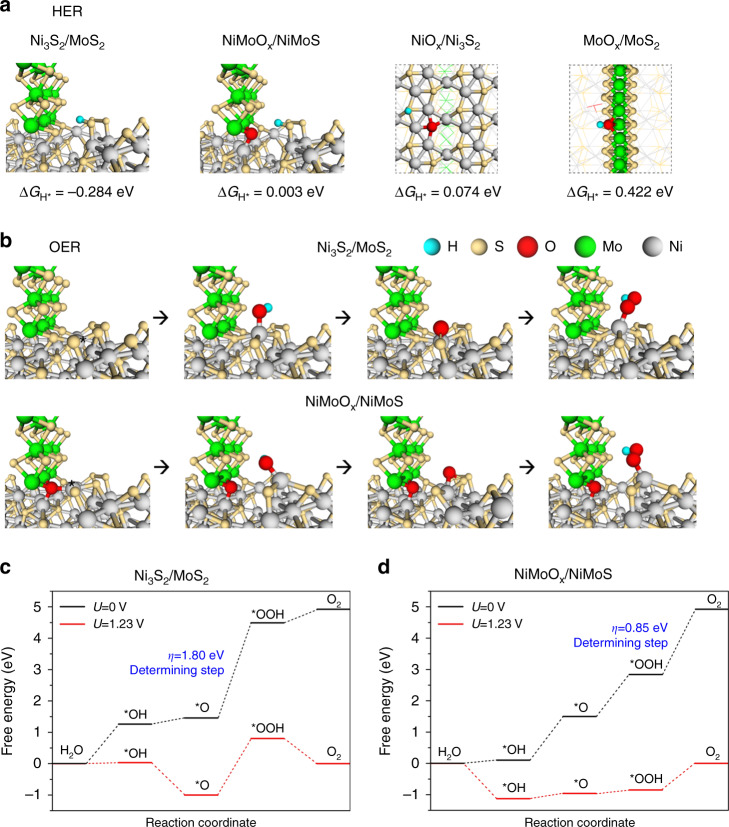
Origin of HER/OER activities on NiMoO_x_/NiMoS. **a** Chemisorption models and corresponding Gibbs free energy of H on the interface of Ni_3_S_2_/MoS_2_ and NiMoO_x_/NiMoS, on the surface of NiO_x_/Ni_3_S_2_ (S) and on the edge of MoO_x_/MoS_2_ (Mo). **b** OH, O, and OOH intermediates adsorption configurations for OER on the interface of (top) Ni_3_S_2_/MoS_2_ and (bottom) NiMoO_x_/NiMoS. **c**, **d** The free energy diagrams for OER on the interface of **c** Ni_3_S_2_/MoS_2_ and (**d**) NiMoO_*x*_/NiMoS heterostructures. Cyan, yellow, red, green, and gray balls, respectively, represents H, S, O, Mo, and Ni atoms.

To explore the original relationship between the intrinsically catalytic activity and the electronic and atomic structures of the interface of NiMoO_*x*_/NiMoS, density functional theory calculations were performed to conduct the Gibbs free energies of every step in HER and OER (Supplementary Fig. 26–33). The hydrogen absorption energy (Δ*G*_H*_) is generally considered as the key descriptor for evaluating the performance of HER^71^. The sulfur sites of NiO_*x*_/Ni_3_S_2_ and MoO_*x*_/MoS_2_ exhibit much lower Δ*G*_H*_ relative to that of Ni_3_S_2_ and MoS_2_ (Fig. 8a and Supplementary Fig. 29–32), indicating that the integration of the oxides and sulfides enables the favorable H* adsorption and the tremendous decrement of thermodynamic barriers for hydrogen production. Especially, the oxidation/hydrogenation-induced surface reconfiguration results into the fabrication of NiMoO_*x*_/NiMoS heterostructure. The sulfur species serve as the distinctive active sites for the optimized hydrogen adsorption with nearly zero Δ*G*_H*_ (0.003 eV), in comparison of NiO_*x*_/Ni_3_S_2_ (Δ*G*_H*_ = 0.074 eV) and MoO_*x*_/MoS_2_ (Δ*G*_H*_ = 0.422 eV). Since oxygen-free NiMoS shows much more negative Δ*G*_H*_ (–0.284 eV) comparing to NiMoO_x_/NiMoS, it is hypothesized that the oxide species of the unique multi-interfaces may avoid the excessively strong adsorption of H* and bring about the facile intermediates desorption. Theoretically, water oxidation in alkaline medium involves four concerted proton-electron transfer steps^72^. The absorption configurations and calculated free energy profiles of OER steps are presented (Figs. 8b-8d). Obviously, the potential rate-determining step (PDS) of NiMoS heterostructures is the third electrochemical step from *O to *OOH with an energy barrier of 1.80 eV. The *OOH species on NiMoO_*x*_/NiMoS heterostructures are greatly stabilized and overpotential is largely reduced to 0.85 V with the PDS of forming molecule O_2_. In comparison of NiMoO_*x*_/NiMoS, it is of noted that the oxide species in NiO_*x*_/Ni_3_S_2_ and MoO_*x*_/MoS_2_ heterointerfaces have small evident impact on the decrement of overpotential (Supplementary Fig. 29–32). Therefore, the multi-interfaces of bimetal oxides/sulfides heterostructures are indispensible for the favorable stabilization of intermediates and accelerated electrochemical kinetics. In order to undestand the charge transfer between the NiMoO_*x*_/NiMoS interface, charge density difference was performed (Supplementary Fig. 33). It is clear that a remarkable charge transfer across the interface, facilitates the fast electron transfer during the electrocatalytic process. Overall, the theory simulations and experiments demonstrate that the excellent OER and HER activities are facilitated by the synergetic effect of the oxidation/hydrogenation-induced surface reconfiguration.

In this case, the robust electrocatalytic activity is firstly ascribed to 3D hierarchical heterostructures of NiMoO_x_/NiMoS array owe to the excellent mass transport and gas permeability. Secondly, the constructed interfaces among various heterostructures not only produce together the activities of different materials, but also facilitate the charge transfer and brings exceptionally synergistic effect of typical catalysts by oxidation/hydrogenation-induced surface reconfiguration strategy. Thirdly, the generation of defective species in hierarchical heterostructures could optimize electric conductivity and generate abundant active sites, confirming by low resistances and large ECSAs. Finally, the synergistic effect of the morphology and heterostructure engineering in NiMoO_x_/NiMoS array promotes the generation of abundant active sites by engineering active sites, optimizing adsorption energies, and accelerating water splitting kinetics. All advantages promote the robust catalytic performance of NiMoO_x_/NiMoS array as a typical catalyst, offering a prospective solution of hierarchical electrocatalysts for practical water splitting applications.

## Discussion

In summary, hierarchical transition bimetal oxides/sulfides array was fabricated by oxidation/hydrogenation-induced surface reconfiguration strategy by use of NiMoS architectures as the precursor, interacting two-dimensional MoO_*x*_/MoS_2_ nanosheets attached to one-dimensional NiO_*x*_/Ni_3_S_2_ nanorods array. To optimize the electrocatalytic performance, the influence of oxygen plasma power and hydrogenation temperature upon HER and OER performance of NiMoO_*x*_/NiMoS array was explored, confirming the best plasma power of 100 W and appropriate thermal treatment temperature at 400 °C. Benefiting from heterostructure engineering, as-synthesized NiMoO_*x*_/NiMoS array presents the remarkable electrocatalytic performance, achieving low overpotentials of 38, 89, 174, and 236 mV for HER and 186, 225, 278, and 334 mV for OER at 10, 100, 500 and 1000 mA cm^−2^, even surviving at large current density of 100 and 500 mA cm^−2^ with long-term stability. The extraordinarily enhanced electrocatalytic performance of transition bimetal oxides/sulfides heterostructure array as the typical model is ascribed to not only the simultaneous modulation of component and geometric structure, but also the systematic optimization of charge transfer, abundant electrocatalytic active sites and exceptionally synergistic effect of heterostructure interfaces. Density functional theory calculations reveal that the coupling interface between NiMoO_x_ and NiMoS optimizes adsorption energies and accelerates water splitting kinetics, thus promoting the catalytic performance. Especially, the assembled two-electrode cell by use of NiMoO_*x*_/NiMoS array delivers the industrially required current densities of 500 and 1000 mA cm^−2^ at record low cell voltages of 1.60 and 1.66 V, along with excellent durability, outperforming most of transition metal-based bifunctional electrocatalysts reported to date. Given hierarchical transition heterostructures array as typical model, this work could open up the avenues to the development of excellent electrocatalysts by engineering active sites for large-scale energy conversion applications.

## Methods

### Materials

Ni foam was purchased from Suzhou Jiashide Metal Foam Co. Ltd. Ni(NO_3_)_2_·6H_2_O, (NH_4_)Mo_7_O_24_·4H_2_O, thiourea and KOH was purchased from Aladdin. Pt/C (20 wt% Pt on Vulcan XC-72R) and Nafion (5 wt%) were purchased from Sigma-Aldrich. All chemicals were used as received without further purification. The water used throughout all experiments was purified through a Millipore system.

### Fabrication of NiMoO_x_/NiMoS heterostructure array

0.07 M Ni(NO_3_)_2_·6H_2_O, 0.01 M (NH_4_)Mo_7_O_24_·4H_2_O and 0.30 M thiourea were dissolved into 15 mL deionized water and stirred for 10 min under room temperature. Then the solution was transferred to a 25 mL Teflon-lined steel autoclave with nickel foam. After hydrothermal reaction at 200 °C for 24 h, NiMoS precursor was obtained through washing with deionized water and then dried in an oven at 60 °C. As-obtained NiMoS precursors were irradiated by RF plasma under an oxygen flow (RF power, 50~150 W) for the oxidation treatment. Afterward the arrays were annealed up to 300–500 °C in H_2_/Ar (0.05/0.95) for the typical hydrogenation regulation, thus resulting into the synthesis of NiMoO_x_/NiMoS heterostructure array by oxidation/hydrogenation-induced surface reconfiguration strategy. In comparison, as-obtained NiO_x_/Ni_3_S_2_ and MoO_x_/MoS_2_ heterostructure arrays were synthesized in parallel by the same procedure as that of NiMoO_x_/NiMoS array expect for in absence of (NH_4_)Mo_7_O_24_·4H_2_O or Ni(NO_3_)_2_·6H_2_O in hydrothermal reaction.

### Structural characterization

Powder XRD patterns of the products were tested with X-ray diffractometer (Japan Rigaku Rotaflex) by Cu K_α_ radiation (*λ* = 1.5418 nm, 40 kV, 40 mA) at room temperature. SEM images of the products were captured by a field-emission scanning electron microscope (SEM, FEI Nova Nano SEM 450). TEM images of the products were performed on transmission electron microscopy (TEM, FEI TF30). The chemical states of the samples were determined by XPS in a Thermo VG ESCALAB250 surface analysis system. The shift of binding energy due to relative surface charging was corrected using the C 1 s level at 284.6 eV as an internal standard.

### Electrochemical measurements

The electrocatalytic HER and OER performance of different electrocatalysts (1 cm^2^) were evaluated using a typical three-electrode system in N_2_ and O_2_-saturated 1 M KOH electrolyte, respectively. All polarization curves at 1 mV s^−1^ were corrected with iR compensation. The mass loading of NiMoS-based electrocatalysts was tested according to the mass difference. Commercial IrO_2_ or 20 wt% Pt/C was dispersed in ethanol solution with Nafion and then the ink was dropped by a micropipettor on Ni foam. The EIS tests were measured by AC impedance spectroscopy at the frequency ranges 10^6^ to 0.1 Hz. According to the Nernst equation (*E*_RHE_ = *E*_Hg/HgO_ + 0.059 pH + 0.098), where *E*_RHE_ was the potential vs. a reversible hydrogen potential, *E*_Hg/HgO_ was the potential vs. Hg/HgO electrode, and pH was the pH value of electrolyte. To determination of Faradaic efficiency, the Faradaic efficiency of HER or OER catalyst is defined as the ratio of the amount of experimentally determined hydrogen or oxygen to that of the theoretically expected hydrogen or oxygen from the HER or OER reaction in 1 M KOH aqueous solution by use of an online gas chromatography system (GC, Techcomp GC 7890 T, Ar carrier gas, Thermo Conductivity Detector). As for the theoretical value, we assumed that 100% current efficiency during the reaction, which means only the HER or OER process was occurring at the working electrode. The theoretically expected amount of hydrogen or oxygen was then calculated by applying the Faraday law, which states that the passage of 96485.4 C causes 1 equivalent of reaction.

### First-principle calculations

Density functional theory calculations were carried out by the Vienna ab initio simulation package (VASP), using the planewave basis with an energy cutoff of 400 eV, the projector augmented wave pseudopotentials, and the generalized gradient approximation parameterized by Perdew, Burke, and Ernzerhof (GGA-PBE) for exchange-correlation functional^73^. The Brillouin zones of the supercells were sampled by 4 × 4 × 1 uniform k point mesh. With fixed cell parameters, the model structures were fully optimized using the convergence criteria of 10^−5^ eV for the electronic energy and 10^−2^ eV/Å for the forces on each atom. The supercells dimension in x and y was 11.598 Å and 12.243 Å, respectively. The vacuum region in the z direction was adopted large than 15 Å so that the spurious interactions of neighboring models are negligible. Then O atom was used to replace the S atom on the edge of MoS_2_ and the surface of Ni_3_S_2_ and the interface of MoS_2_ and Ni_3_S_2_, respectively^74^. To simulate the edge, the surface and interface incorporate with the oxides. Both spin-polarized and spin-unpolarized computations were performed. The computational results show that both NiMoS and NiMoO_x_/NiMoS are magnetic. In addition, we applied the DFT-D3 (BJ) method to evaluate the van der Waals (vdW) effect in all calculations.

The Gibbs free energy of the intermediates for HER and OER process, that is, H, OH, O, and OOH, can be calculated as^75,76^

1 $${\Delta} G = E_{{\rm{ads}}} + {\Delta} E_{{\rm{ZPE}}} - T{\Delta} S$$

where *E*_ads_ is the adsorption energy of intermediate, Δ*E*_ZPE_ is the zero point energy difference between the adsorption state and gas state, *T* is the temperature, and Δ*S* is the entropy various between the adsorption and gas phase. For adsorbates, *E*_ZPE_ and *S* are obtained from vibrational frequencies calculations with harmonic approximation and contributions from the slabs are neglected, whereas for molecules these values are taken from NIST-JANAF thermochemical Tables^77^. The contributions are listed (Supplementary Table 8). Usually, the vibration entropy of hydrogen adsorption on the substrate is small, the entropy of hydrogen adsorption  is ΔS ≈ −1/2*S*°, where *S*^0^ is the entropy of H_2_ in the gas phase at the standard conditions. The corrected for free energy equation was defined by

2 $${\Delta} G = E_{{\rm{ads}}} + 0.24\,{\rm{eV}}$$

The intermediates adsorption energy *E*_ads_ for *H, *OH, *O, and *OOH can be used as DFT ground state energy calculated as

3 $${\Delta} E_{{{{\,}^ \ast {\rm{H}}}}} = E( {{{{\,}^\ast{\rm{H}}}}} ) - E( \ast ) - 1/2E( {{\rm{H}}_2} )$$

4 $${\Delta} E_{{{{\,}^ \ast {\rm{OOH}}}}} = E( {{{{\,}^ \ast {\rm{OOH}}}}} ) - E( \ast ) - ( {2E_{{\rm{H}}_2{\rm{O}}} - 3/2E_{{\rm{H}}_2}} )$$

5 $${\Delta} E_{{{{\,}^ \ast {\rm{O}}}}} = E( {{{{\,}^ \ast {\rm{O}}}}} ) - E( \ast ) - ( {E_{{\rm{H}}_2{\rm{O}}} - E_{{\rm{H}}_2}} )$$

6 $${\Delta} E_{{{{\,}^\ast {\rm{OH}}}}} = E( {{{{\,}^ \ast {\rm{OH}}}}} ) - E( \ast ) - ( {E_{{\rm{H}}_2{\rm{O}}} - 1/2E_{{\rm{H}}_2}} )$$

The OER process in alkaline medium generally occur through the following steps:

7 $$\ast + {\rm{OH}}^ - \to {\rm{OH}}^ \ast + {\rm{e}}^ - \quad\quad\quad\quad\quad\quad\quad {\Delta} G_1$$

8 $${\rm{OH}}^ \ast + {\rm{OH}}^ - \to ^\ast {\rm{O}} + {\rm{H}}_2{\rm{O}} + {\rm{e}}^ -\quad \quad\quad\quad {\Delta} G_2$$

9 $${{{\,}^\ast {\rm{O}}}} + {\rm{OH}}^ - \to {\rm{OOH}}^ \ast + {\rm{e}}^ - \quad\quad\quad\quad\quad\quad{\Delta} G_3$$

10 $${{{\rm{OOH}}^\ast}} + {\rm{OH}}^ - \to {\rm{O}}_2 + {\rm{H}}_2{\rm{O}} + {\rm{e}}^ - + \ast \quad\quad {\Delta} G_4$$

where * denotes adsorption active site on the substrate.

11 $${\Delta} G_1 = {\Delta} G_{{\,}^{{\ast{\rm{OH}}}}}$$

12 $${\Delta} G_2 = {\Delta} G_{{{{\,}^ \ast {\rm{O}}}}} - {\Delta} G_{{{{\,}^ \ast {\rm{OH}}}}}$$

13 $${\Delta} G_3 = {\Delta} G_{{{{\,}^ \ast {\rm{OOH}}}}} - {\Delta} G_{{{{\,}^ \ast {\rm{O}}}}}$$

14 $${\Delta} G_4 = 4.92 - {\Delta} G_{{{{\,}^ \ast {\rm{OOH}}}}}$$

The overpotential η is defined as

15 $$\eta = \max \left\{ {{\Delta} G_1,{\Delta} G_2,{\Delta} G_3,{\Delta} G_4} \right\} - 1.23\,{\rm{eV}}$$

## Supplementary information

Supporting Information

Peer Review File

Supplementary Video

## Data Availability

The data that support the findings of this work are available from the corresponding author upon reasonable request.

## References

[1] Jiao Y (2015). Design of electrocatalysts for oxygen- and hydrogen-involving energy conversion reactions. Chem. Soc. Rev..

[2] Xia Z, Guo S (2019). Strain engineering of metal-based nanomaterials for energy electrocatalysis. Chem. Soc. Rev..

[3] Wei C (2019). Approaches for measuring the surface areas of metal oxide electrocatalysts for determining their intrinsic electrocatalytic activity. Chem. Soc. Rev..

[4] Zou X, Zhang Y (2015). Noble metal-free hydrogen evolution catalysts for water splitting. Chem. Soc. Rev..

[5] Pi Y (2017). Trimetallic oxyhydroxide coralloids for efficient oxygen evolution electrocatalysis. Angew. Chem. Int. Ed..

[6] Huang L (2019). Zirconium-regulation-induced bifunctionality in 3D cobalt-iron oxide nanosheets for overall water splitting. Adv. Mater..

[7] Hao S (2019). NiCoMo hydroxide nanosheet arrays synthesized via chloride corrosion for overall water splitting. ACS Energy Lett..

[8] Hou J (2017). Active sites intercalated ultrathin carbon sheath on nanowire arrays as integrated core-shell architecture: highly efficient and durable electrocatalysts for overall water splitting. Small.

[9] Yang L (2018). Efficient oxygen evolution electrocatalysis in acid by a perovskite with face-sharing IrO_6_ octahedral dimmers. Nat. Commun..

[10] Chen H (2019). Promoting subordinate, efficient ruthenium sites with interstitial silicon for Pt-Like electrocatalytic activity. Angew. Chem. Int. Ed..

[11] Liang X (2019). Activating inert, nonprecious perovskites with iridium dopants for efficient oxygen evolution reaction under acidic conditions. Angew. Chem. Int. Ed..

[12] Huang LB (2018). Self-limited on-site conversion of MoO_3_ nanodots into vertically aligned ultrasmall monolayer MoS_2_ for efficient hydrogen evolution. Adv. Energy Mater..

[13] Zhang J (2017). Efficient hydrogen production on MoNi_4_ electrocatalysts with fast water dissociation kinetics. Nat. Commun..

[14] Chen YY (2017). Self-templated fabrication of MoNi_4_/MoO_3-x_ nanorod arrays with dual active components for highly efficient hydrogen evolution. Adv. Mater..

[15] An L (2019). Epitaxial heterogeneous interfaces on N-NiMoO_4_/NiS_2_ nanowires/nanosheets to boost hydrogen and oxygen production for overall water splitting. Adv. Funct. Mater..

[16] Zhang X (2018). Co_3_O_4_/Fe_0.33_Co_0.66_P interface nanowire for enhancing water oxidation catalysis at high current density. Adv. Mater..

[17] Liu T (2018). Interfacial electron transfer of Ni_2_P-NiP_2_ polymorphs inducing enhanced electrochemical properties. Adv. Mater..

[18] Niu S (2019). Autogenous growth of hierarchical NiFe(OH)_x_/FeS nanosheet‐on‐microsheet arrays for synergistically enhanced high‐output water oxidation. Adv. Funct. Mater..

[19] Peng YW (2019). Polyoxometalate‐derived ultrasmall Pt_2_W/WO_3_ heterostructure outperforms platinum for large‐current‐density H_2_ evolution. Adv. Energy Mater..

[20] Hou J (2018). Promoting active sites in core-shell nanowire array as Mott-Schottky electrocatalysts for efficient and stable overall water splitting. Adv. Funct. Mater..

[21] Feng JX (2015). Co(OH)_2_@PANI hybrid nanosheets with 3D networks as high-performance electrocatalysts for hydrogen evolution reaction. Adv. Mater..

[22] Feng JX (2016). FeOOH/Co/FeOOH hybrid nanotube arrays as high-performance electrocatalysts for the oxygen evolution reaction. Angew. Chem. Int. Ed..

[23] Lei C (2019). Efficient alkaline hydrogen evolution on atomically dispersed Ni–N_x_ species anchored porous carbon with embedded Ni nanoparticles by accelerating water dissociation kinetics. Energy Environ. Sci..

[24] Yan H (2019). Anion-modulated HER and OER activities of 3D Ni-V-based interstitial compound heterojunctions for high-efficiency and stable overall water splitting. Adv. Mater..

[25] Hou J (2018). Electrical behavior and electron transfer modulation of nickel-copper nanoalloys confined in nickel-copper nitrides nanowires array encapsulated in nitrogen-doped carbon framework as robust bifunctional electrocatalyst for overall water splitting. Adv. Funct. Mater..

[26] Yoon T, Kim KS (2016). One-step synthesis of CoS-Doped β-Co(OH)_2_@amorphous MoS_2+x_ hybrid catalyst grown on nickel foam for high-performance electrochemical overall water splitting. Adv. Funct. Mater..

[27] Wu Y (2018). Coupling interface constructions of MoS_2_/Fe_5_Ni_4_S_8_ heterostructures for efficient electrochemical water splitting. Adv. Mater..

[28] Zhang J (2016). Interface engineering of MoS_2_/Ni_3_S_2_ heterostructures for highly enhanced electrochemical overall-water-splitting activity. Angew. Chem. Int. Ed..

[29] An T (2016). Interlaced NiS_2_–MoS_2_ nanoflake-nanowires as efficient hydrogen evolution electrocatalysts in basic solutions. J. Mater. Chem. A.

[30] Yang Y (2019). Hierarchical nanoassembly of MoS_2_/Co_9_S_8_/Ni_3_S_2_/Ni as a highly efficient electrocatalyst for overall water splitting in a wide pH range. J. Am. Chem. Soc..

[31] Li H (2018). Systematic design of superaerophobic nanotube-array electrode comprised of transition-metal sulfides for overall water splitting. Nat. Commun..

[32] Wang X (2020). Single-atom vacancy defect to trigger high-efficiency hydrogen evolution of MoS_2_. J. Am. Chem. Soc..

[33] Luo Z (2020). Reactant friendly hydrogen evolution interface based on di-anionic MoS_2_ surface. Nat. Commun..

[34] He W (2019). Fluorine-anion-modulated electron structure of nickel sulfide nanosheet arrays for alkaline hydrogen evolution. ACS Energy Lett..

[35] Feng L (2015). High-index faceted Ni_3_S_2_ nanosheet arrays as highly active and ultrastable electrocatalysts for water splitting. J. Am. Chem. Soc..

[36] Zhu H (2015). When cubic cobalt sulfide meets layered molybdenum disulfide: A core–shell system toward synergetic electrocatalytic water splitting. Adv. Mater..

[37] Feng J (2018). Efficient hydrogen evolution on Cu nanodots-decorated Ni_3_S_2_ nanotubes by optimizing atomic hydrogen adsorption and desorption. J. Am. Chem. Soc..

[38] Chen D (2018). Preferential cation vacancies in perovskite hydroxide for the oxygen evolution reaction. Angew. Chem. Int. Ed..

[39] Xiao Z (2017). Filling the oxygen vacancies in Co_3_O_4_ with phosphorus: an ultra-efficient electrocatalyst for overall water splitting. Energy Environ. Sci..

[40] Wang Y (2018). 3D carbon electrocatalysts in situ constructed by defect-rich nanosheets and polyhedrons from NaCl-sealed zeolitic imidazolate frameworks. Adv. Funct. Mater..

[41] Smith R (2013). Photochemical route for accessing amorphous metal oxide materials for water oxidation catalysis. Science.

[42] Hou J (2018). Vertically aligned oxygenated-CoS_2_–MoS_2_ heteronanosheet architecture from polyoxometalate for efficient and stable overall water splitting. ACS Catal..

[43] Meng L (2018). Simultaneous manipulation of O-doping and metal vacancy in atomically thin Zn_10_In_16_S_34_ nanosheet arrays toward improved photoelectrochemical performance. Angew. Chem. Int. Ed..

[44] Li H (2015). Activating and optimizing MoS_2_ basal planes for hydrogen evolution through the formation of strained sulphur vacancies. Nat. Mater..

[45] Kibsgaard J, Jaramillo TF (2014). Molybdenum phosphosulfide: An active, acid-Stable, earth-abundant catalyst for the hydrogen evolution reaction. Angew. Chem. Int. Ed..

[46] Kibsgaard J (2015). Designing an improved transition metal phosphide catalyst for hydrogen evolution using experimental and theoretical trends. Energy Environ. Sci..

[47] Han X (2018). Ultrafine Pt nanoparticle-decorated pyrite-type CoS_2_ nanosheet arrays coated on carbon cloth as a bifunctional electrode for overall water splitting. Adv. Energy Mater..

[48] Liu Y (2018). Corrosion engineering towards efficient oxygen evolution electrodes with stable catalytic activity for over 6000 hours. Nat. Commun..

[49] Hao W (2020). Fabrication of practical catalytic electrodes using insulating and eco-friendly substrates for overall water splitting. Energy Environ. Sci..

[50] Wang D (2019). Atomic and electronic modulation of self-supported nickel-vanadium layered double hydroxide to accelerate water splitting kinetics. Nat. Commun..

[51] Zhang J (2019). Modulation of inverse spinel Fe_3_O_4_ by phosphorus doping as an industrially promising electrocatalyst for hydrogen evolution. Adv. Mater..

[52] Zhu C (2018). Fe-Ni-Mo nitride porous nanotubes for full water splitting and Zn-air batteries. Adv. Energy Mater..

[53] Sun Y (2018). Strong electronic interaction in dual-cation-incorporated NiSe_2_ nanosheets with lattice distortion for highly efficient overall water splitting. Adv. Mater..

[54] Lin J (2019). Defect-rich heterogeneous MoS_2_/NiS_2_ nanosheets electrocatalysts for efficient overall water splitting. Adv. Sci..

[55] Zhai Z (2018). Dimensional construction and morphological tuning of heterogeneous MoS_2_/NiS electrocatalysts for efficient overall water splitting. J. Mater. Chem. A.

[56] Wang Z (2018). Phosphorus-doped Co_3_O_4_ nanowire array: a highly efficient bifunctional electrocatalyst for overall water splitting. ACS Catal..

[57] Li M (2019). Ni strongly coupled with Mo_2_C encapsulated in nitrogen-doped carbon nanofibers as robust bifunctional catalyst for overall water splitting. Adv. Energy Mater..

[58] Li S (2016). Co-Ni-based nanotubes/nanosheets as efficient water splitting electrocatalysts. Adv. Energy Mater..

[59] Sivanantham A, Ganesan P, Shanmugam S (2016). Hierarchical NiCo_2_S_4_ nanowire arrays supported on Ni foam: an efficient and durable bifunctional electrocatalyst for oxygen and hydrogen evolution reactions. Adv. Funct. Mater..

[60] Liu B (2018). Iron vacancies induced bifunctionality in ultrathin feroxyhyte nanosheets for overall water splitting. Adv. Mater..

[61] Ledendecker M (2015). The synthesis of nanostructured Ni_5_P_4_ films and their use as a non-noble bifunctional electrocatalyst for full water splitting. Angew. Chem. Int. Ed..

[62] Yan X (2016). From water oxidation to reduction: transformation from Ni_x_Co_3-x_O_4_ nanowires to NiCo/NiCoO_x_ heterostructures. ACS Appl. Mater. Interfaces.

[63] Zhao X (2018). Bifunctional electrocatalysts for overall water splitting from an iron/nickel-based bimetallic metal-organic framework/dicyandiamide composite. Angew. Chem. Int. Ed..

[64] Yang Y (2015). Porous cobalt-based thin film as a bifunctional catalyst for hydrogen generation and oxygen generation. Adv. Mater..

[65] Yu L (2019). Non-noble metal-nitride based electrocatalysts for high-performance alkaline seawater electrolysis. Nat. Commun..

[66] Li H (2019). Amorphous nickel-cobalt complexes hybridized with 1T-phase molybdenum disulfide via hydrazineinduced phase transformation for water splitting. Nat. Commun..

[67] Hui L (2018). Overall water splitting by graphdiyne-exfoliated and -sandwiched layered double-hydroxide nanosheet arrays. Nat. Commun..

[68] Wang Q (2020). Coordination engineering of iridium nanocluster bifunctional electrocatalyst for highly efficient and pH-universal overall water splitting. Nat. Commun..

[69] Shi H (2020). Spontaneously separated intermetallic Co_3_Mo from nanoporous copper as versatile electrocatalysts for highly efficient water splitting. Nat. Commun..

[70] Yu F (2018). High-performance bifunctional porous non-noble metal phosphide catalyst for overall water splitting. Nat. Commun..

[71] Nørskov JK (2005). Trends in the exchange current for hydrogen evolution. J. Electrochem. Soc..

[72] Koper MTM (2013). Theory of multiple proton–electron transfer reactions and its implications for electrocatalysis. Chem. Sci..

[73] Perdew JP, Burke K, Ernzerhof M (1996). Generalized gradient approximation made simple. Phys. Rev. Lett..

[74] Peng L (2019). Rationally design of monometallic NiO-Ni_3_S_2_/NF heteronanosheets as bifunctional electrocatalysts for overall water splitting. J. Catal..

[75] Zhang B (2020). Integrating Rh species with NiFe-layered double hydroxide for overall water splitting. Nano Lett..

[76] Rossmeisl J, Logadottir A, Nørskov JK (2005). Electrolysis of water on (oxidized) metal surfaces. Chem. Phys..

[77] NIST-JANAF Thermochemical Tables. https://janaf.nist.gov/.

