# Supplementary material for: Engineering active sites on hierarchical transition bimetal oxides/sulfides heterostructure array enabling robust overall water splitting
Source: Nat Commun. 2020 Oct 29;11:5462. doi: 10.1038/s41467-020-19214-w (PMC7596725; doi:10.1038/s41467-020-19214-w)
Supplement: Supplementary file 1 — Supporting Information [file 41467_2020_19214_MOESM1_ESM.pdf]

Supporting Information

Engineering active sites on hierarchical transition bimetal oxides/sulfides heterostructure array enabling robust overall water splitting

Panlong Zhai,[1,5] Yanxue Zhang,[2,5] Yunzhen Wu,[1,5] Junfeng Gao,[2] Bo Zhang,[1] Shuyan Cao,[1] Yanting Zhang,[1] Zhuwei Li,[1] Licheng Sun,[1,3,4] Jungang Hou[1]*

[1]State Key Laboratory of Fine Chemicals, School of Chemical Engineering, Dalian University of Technology, Dalian 116024, P. R. China.
[2]Laboratory of Materials Modification by Laser, Ion and Electron Beams, Dalian University of Technology, Ministry of Education, Dalian 116024, P. R. China.
[3]College of Science, Westlake University, Hangzhou 310024, P. R. China.
[4]Department of Chemistry, School of Engineering Sciences in Chemistry, Biotechnology and Health, KTH Royal Institute of Technology, 10044 Stockholm, Sweden.
[5]These authors contributed equally: Panlong Zhai, Yanxue Zhang and Yunzhen Wu.
*E-mail: jhou@dlut.edu.cn

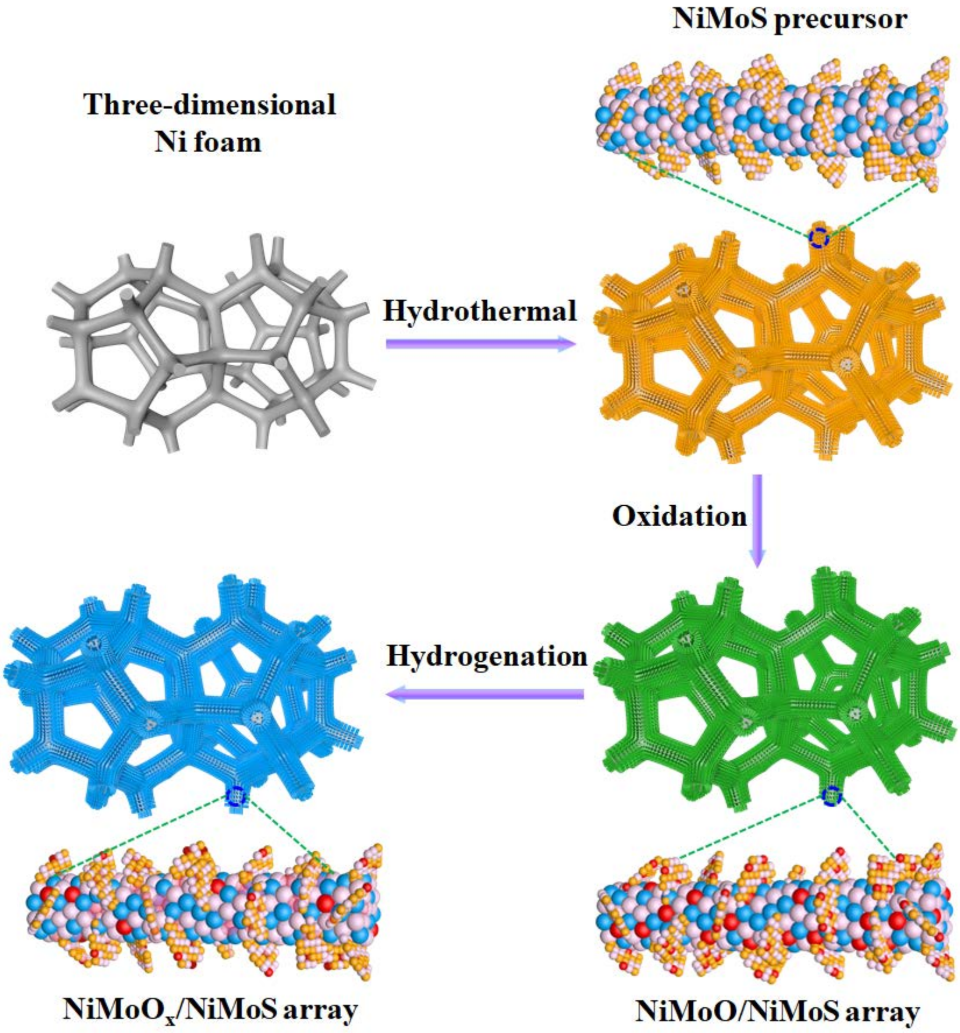

Supplementary Figure 1. Synthesis illustration of NiMoO$_x$/NiMoS heterostructure array by oxidation/hydrogenation-induced surface reconfiguration strategy.

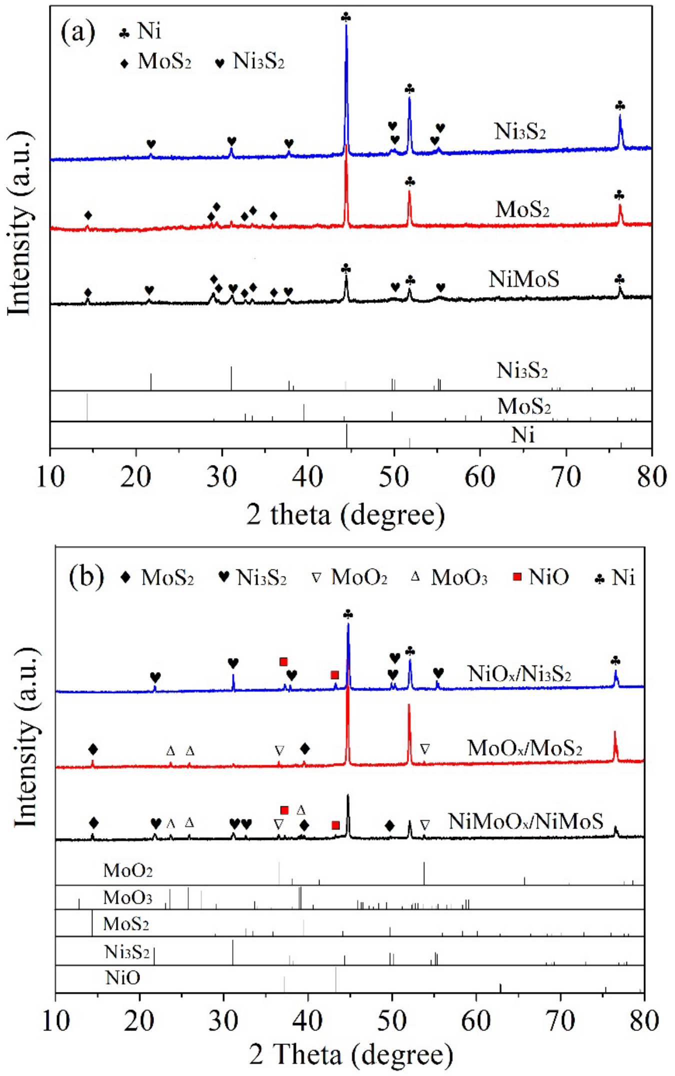

Supplementary Figure 2. XRD patterns of different arrays. (a) MoS_2 and Ni_3S_2 as well as MoS_2/Ni_3S_2, (b) NiO_x/Ni_3S_2, MoO_x/MoS_2 and $NiMoO_x/NiMoS$.

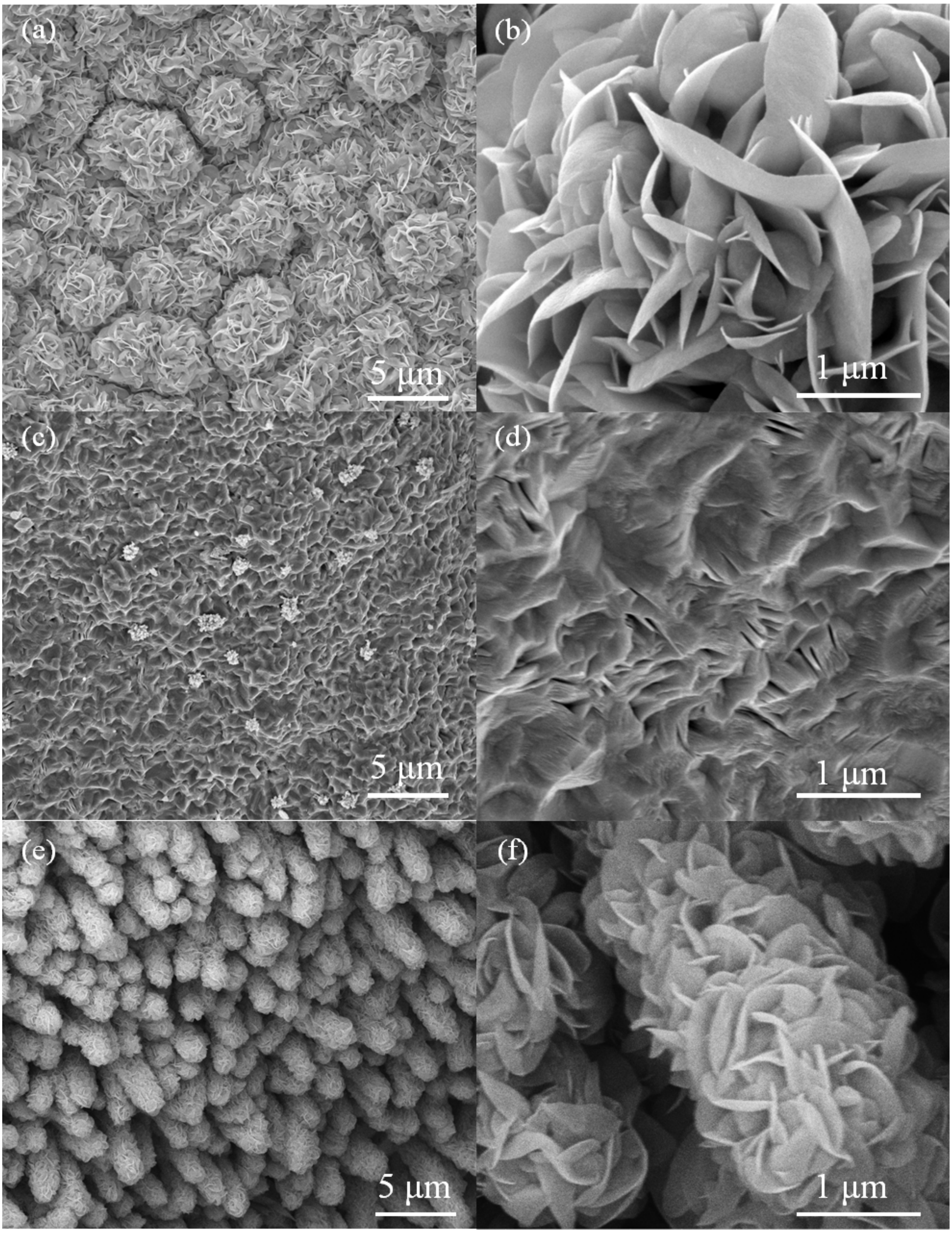

Supplementary Figure 3. SEM images of (ab) MoS_2, (cd) Ni_3S_2 and (ef) NiMoS arrays.

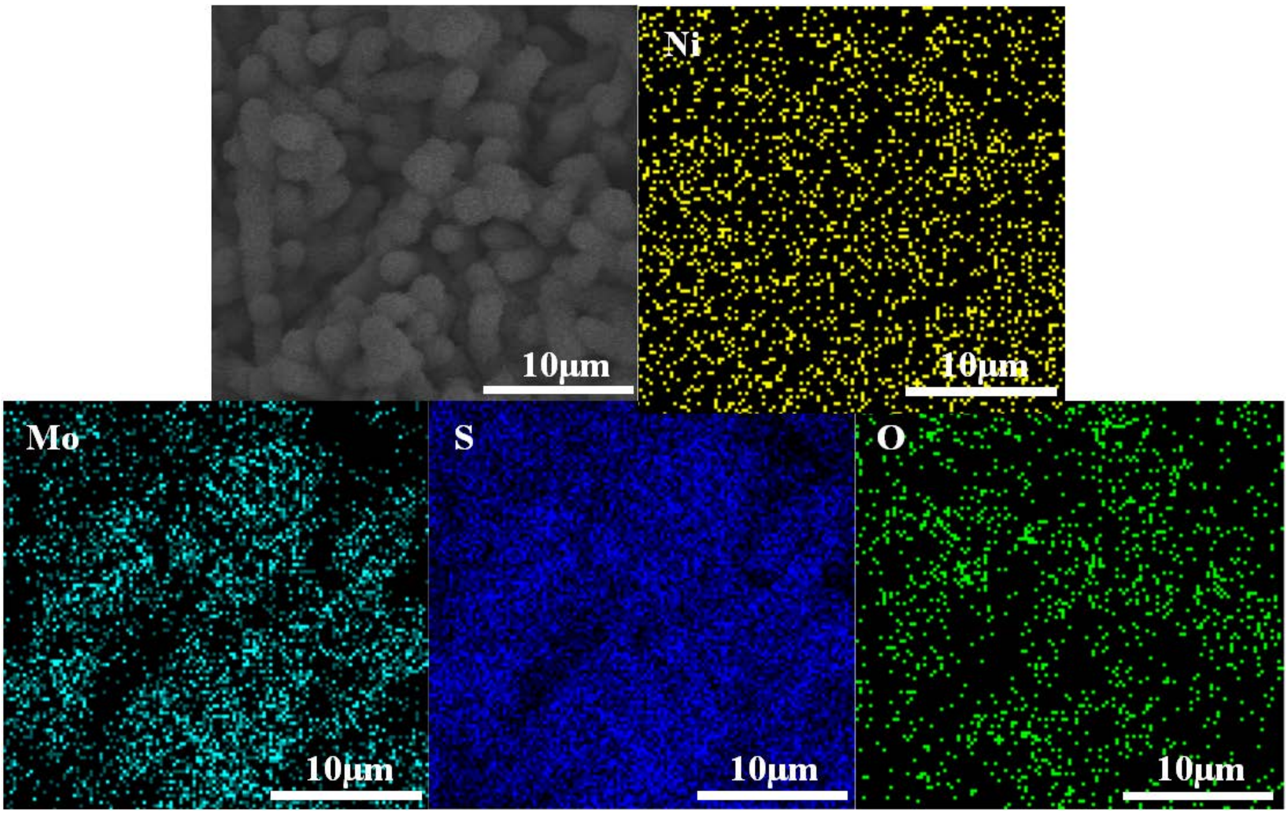

Supplementary Figure 4. Element mapping of $NiMoO_x$/NiMoS heterostructure array by use of oxidation/hydrogenation-induced surface reconfiguration treatment.

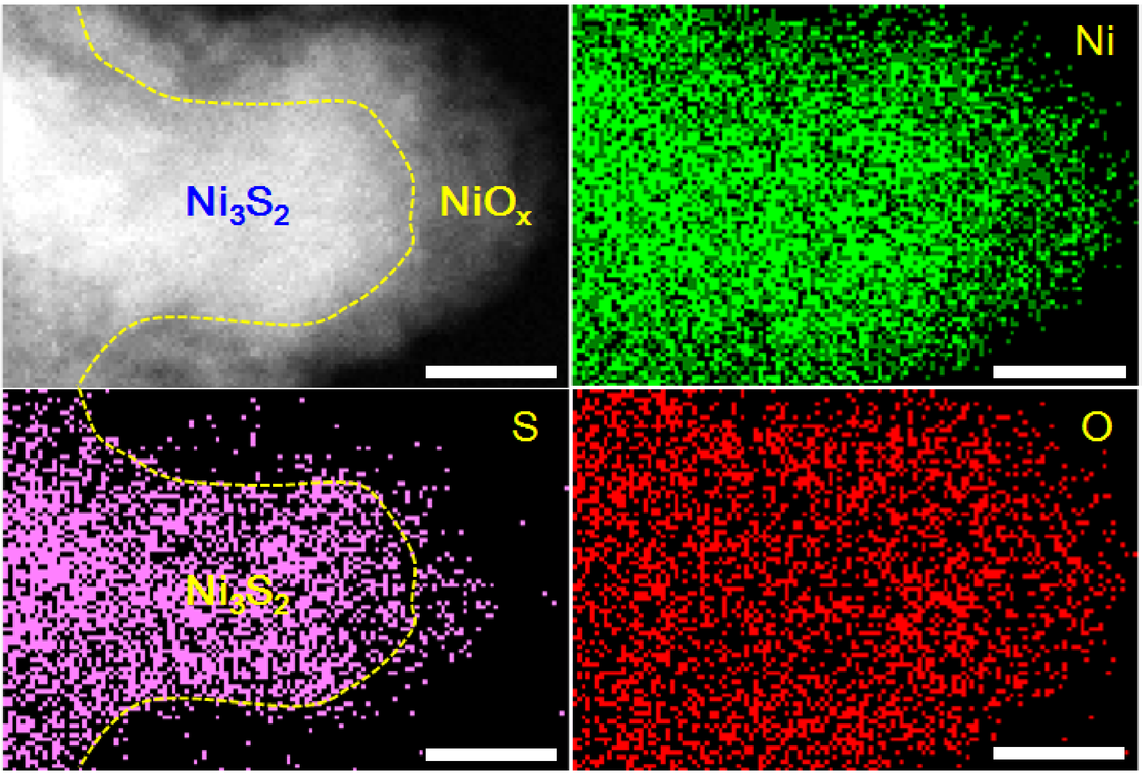

Supplementary Figure 5. HAADF-STEM image and the corresponding EDX elemental mapping of NiO_x/Ni_3S_2 from $NiMoO_x/NiMoS$. Scale bar, 10 nm.

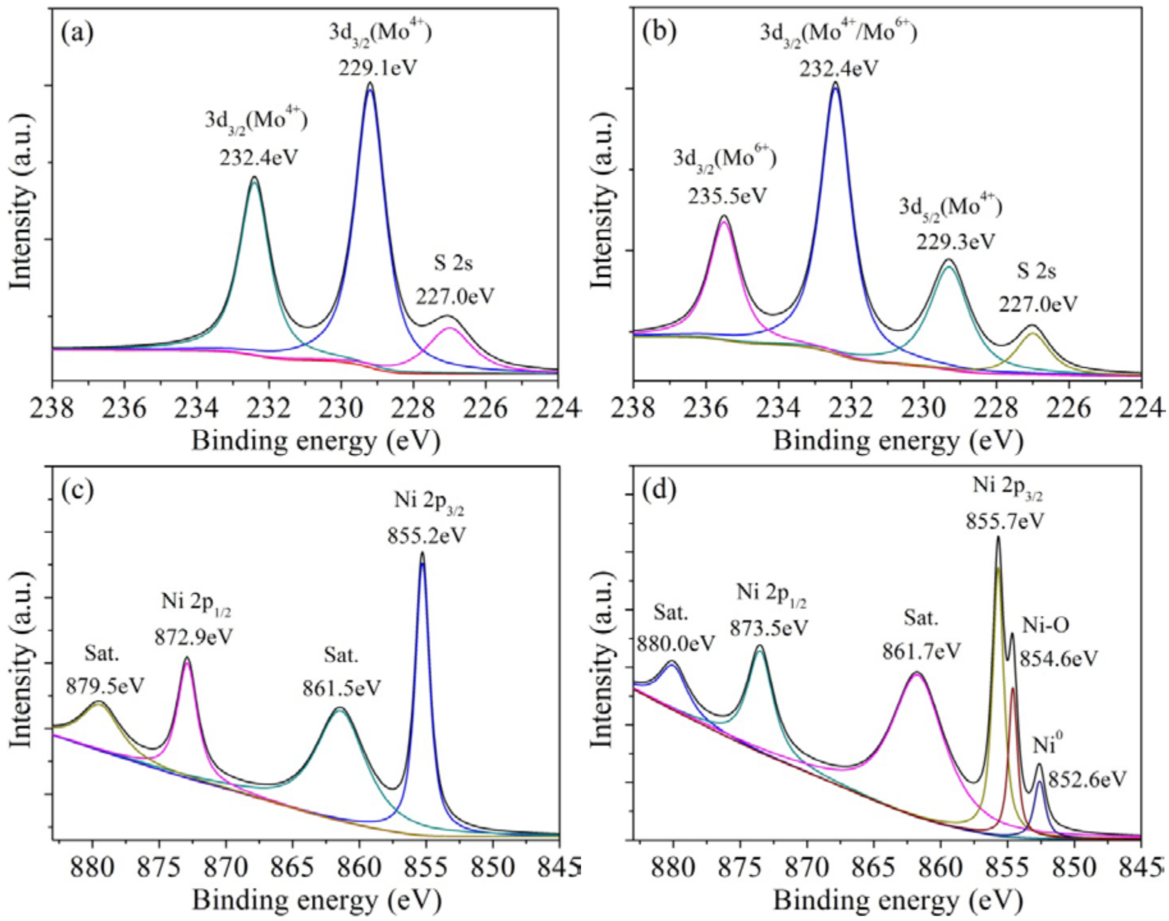

Supplementary Figure 6. XPS spectra of NiMoS and $NiMoO_x$/NiMoS arrays. (ab) Mo 3d and (cd) Ni 2p.

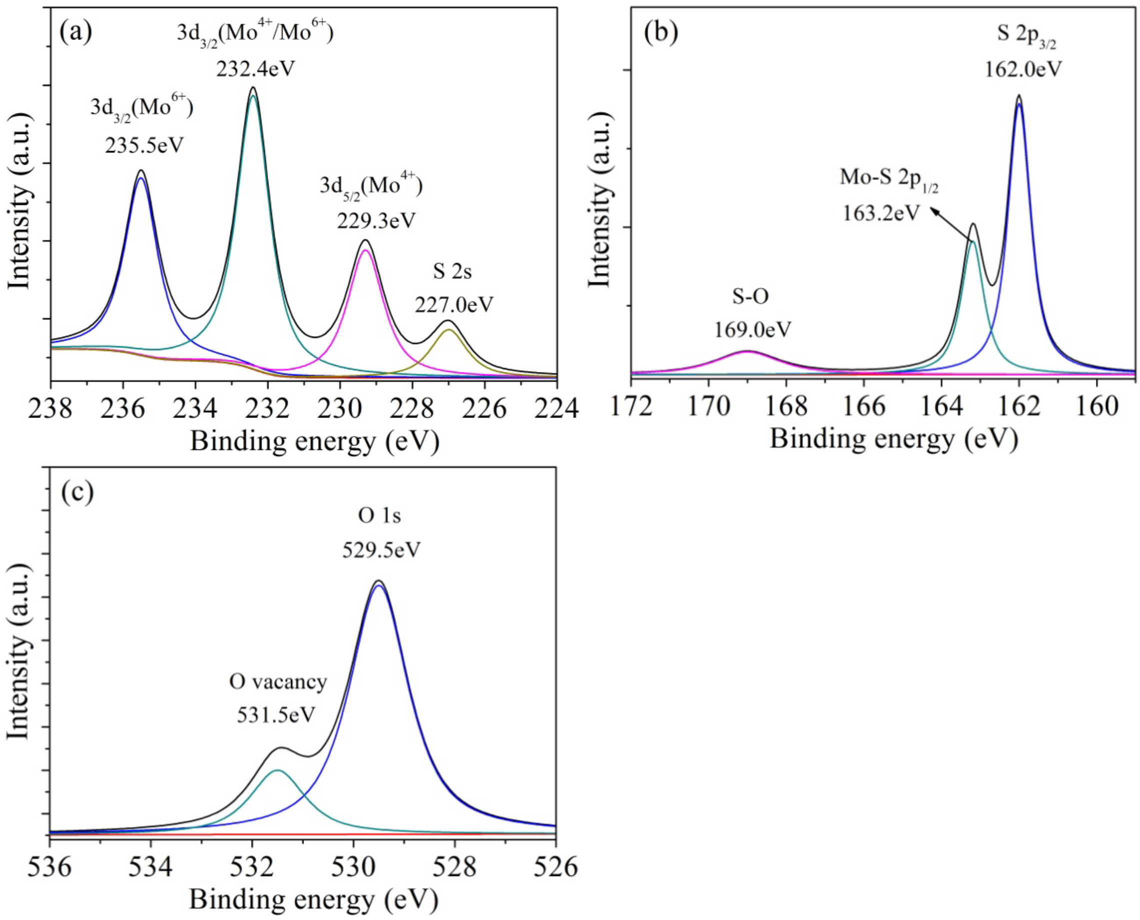

Supplementary Figure 7. XPS spectra of MoO_x/MoS_2 array. (a) Mo 3d, (b) S 2p and (c) O 1s.

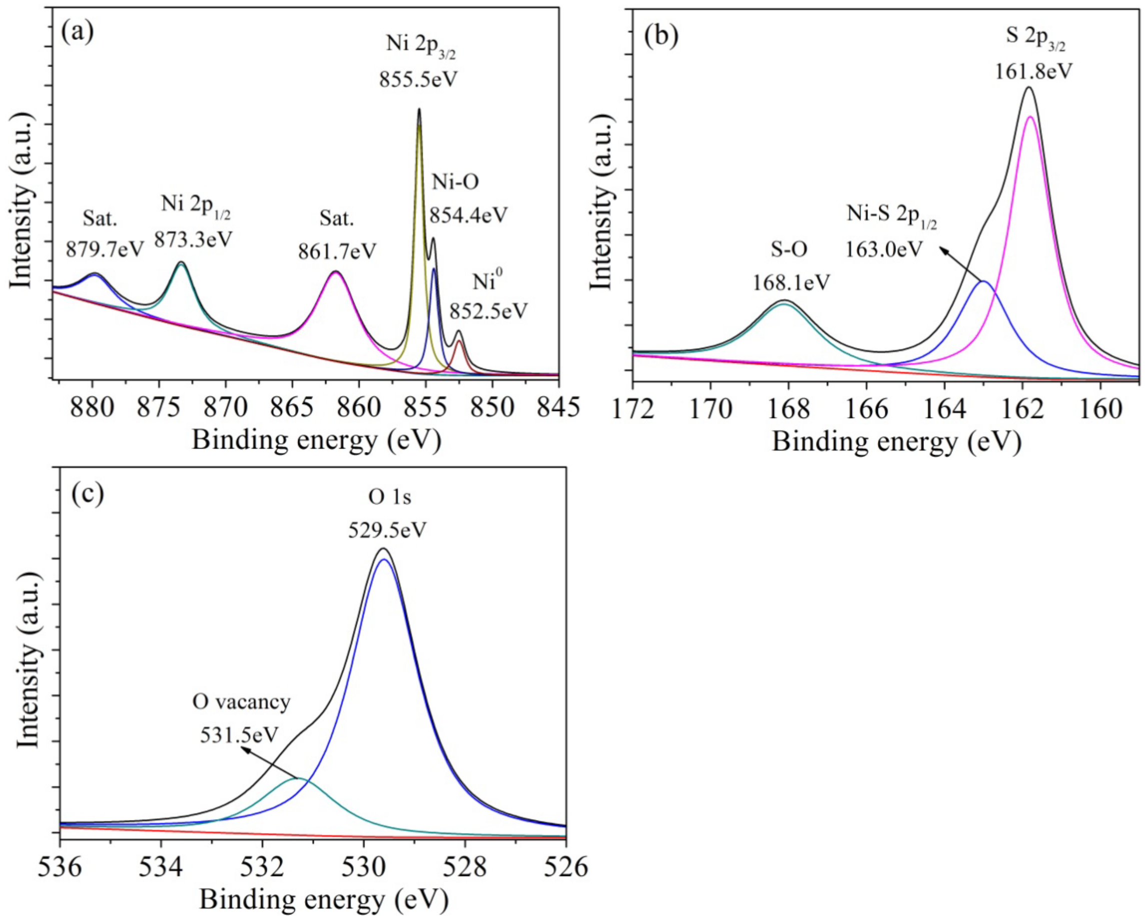

Supplementary Figure 8. XPS spectra of NiO_x/Ni_3S_2 array. (a) Ni 3d, (b) S 2p and (c) O 1s.

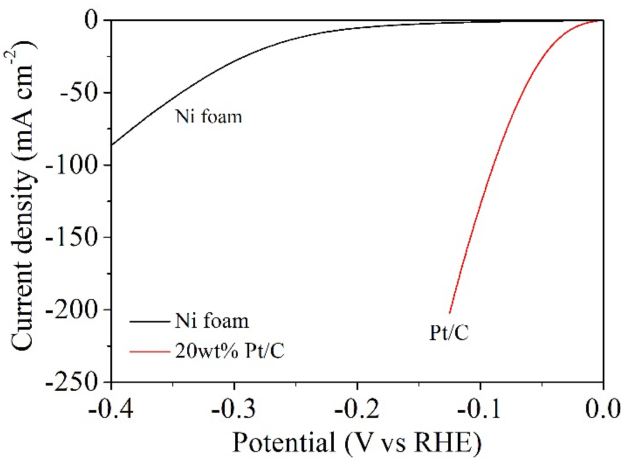

Supplementary Figure 9. HER polarization curves of commercial Pt/C supported on Ni foam in comparison of pristine Ni foam in 1 M KOH.

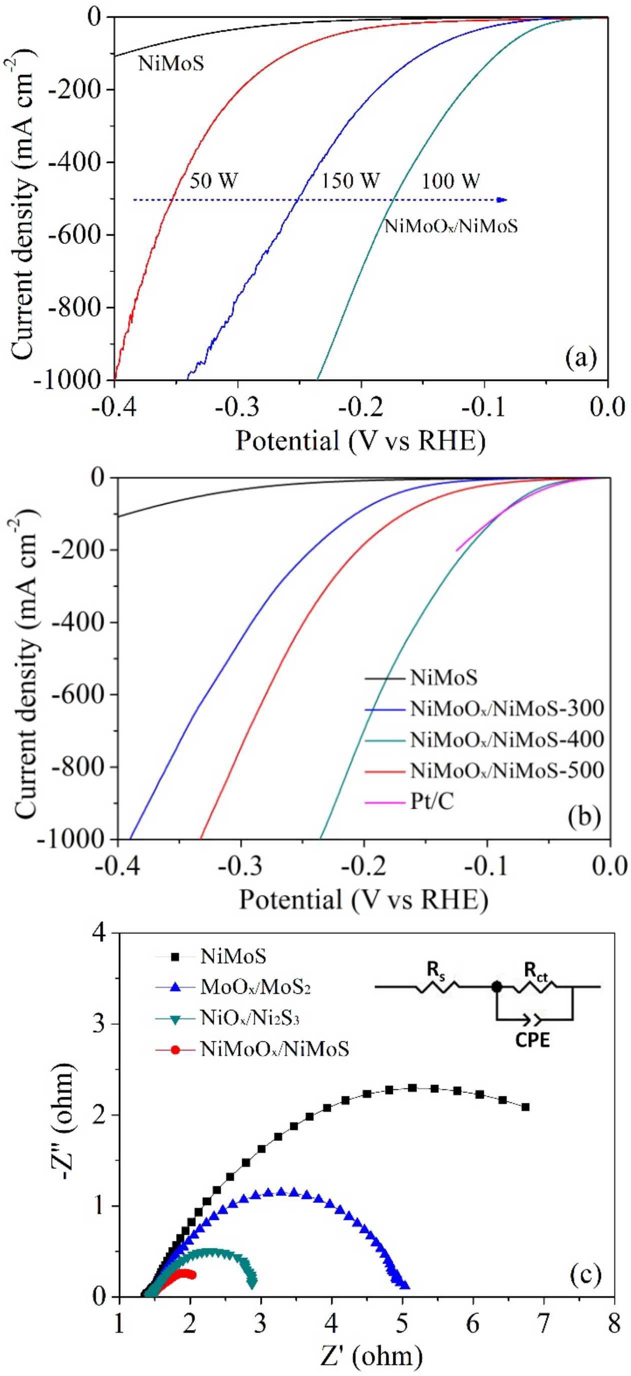

Supplementary Figure 10. (ab) LSV curves of different arrays for HER by tunable power and (b) hydrogenation temperatures, (c) electrochemical impedance spectroscopy tests.

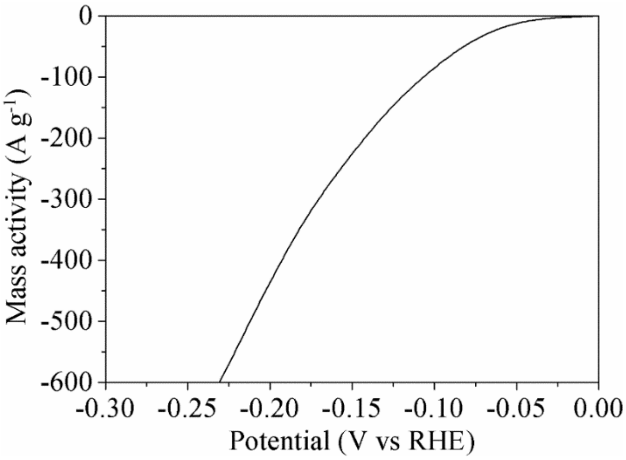

Supplementary Figure 11. Calculated mass activity of $NiMoO_x$/NiMoS array for HER in 1 M KOH.

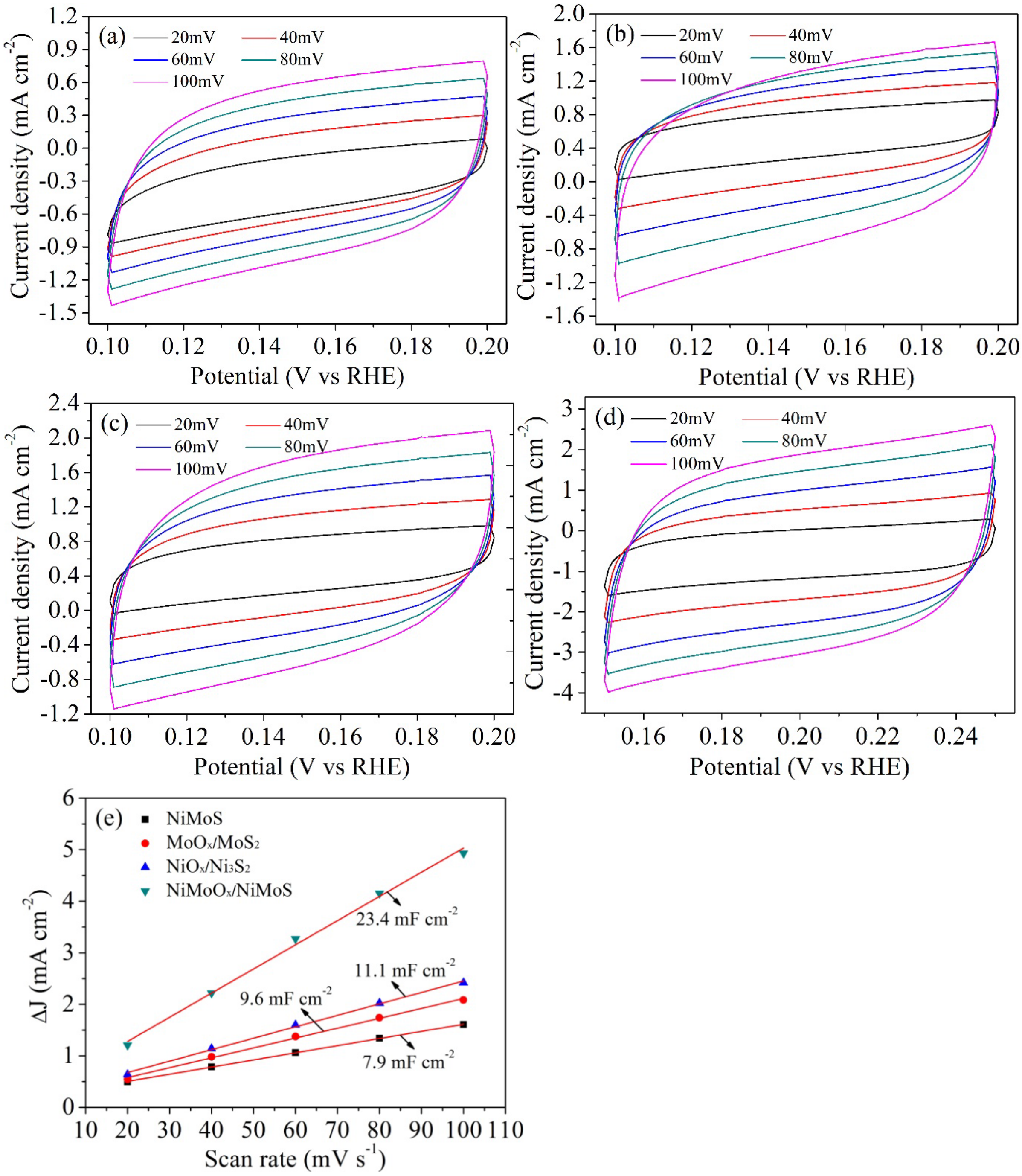

Supplementary Figure 12. (abcd) Cyclic voltammetry curves and (e) electrochemical double-layer capacitances of various catalysts supported on Ni foam during HER process.

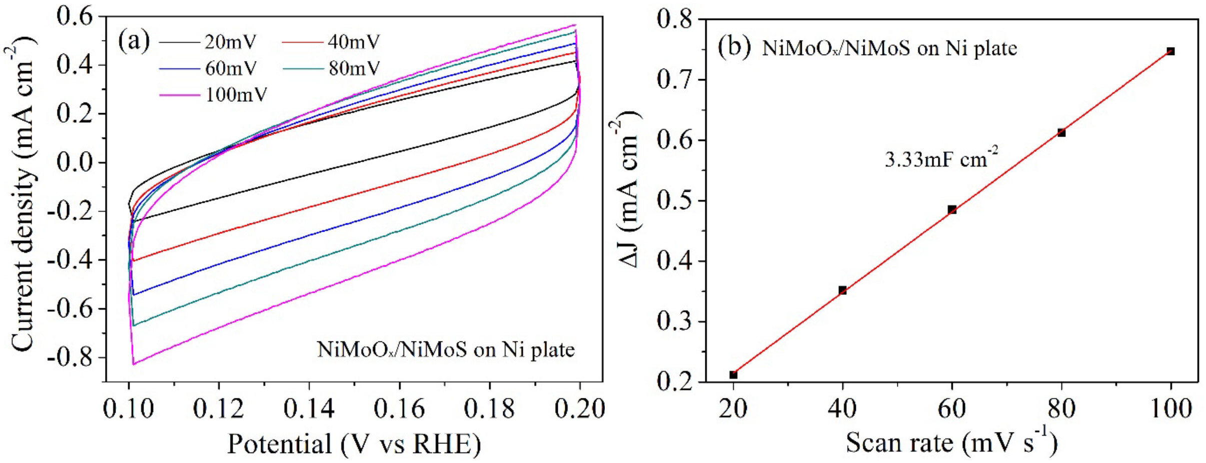

Supplementary Figure 13. (a) Cyclic voltammetry curves and (b) electrochemical double-layer capacitance of $NiMoO_x$/NiMoS supported on Ni plate.

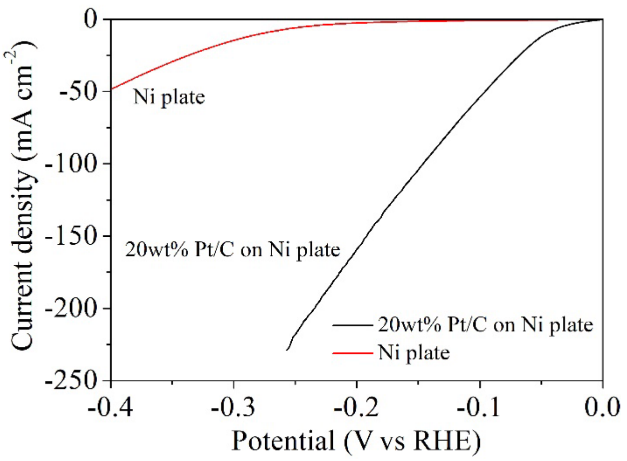

Supplementary Figure 14. HER polarization curves of commercial Pt/C supported on Ni plate in comparison of pristine Ni plate in 1 M KOH.

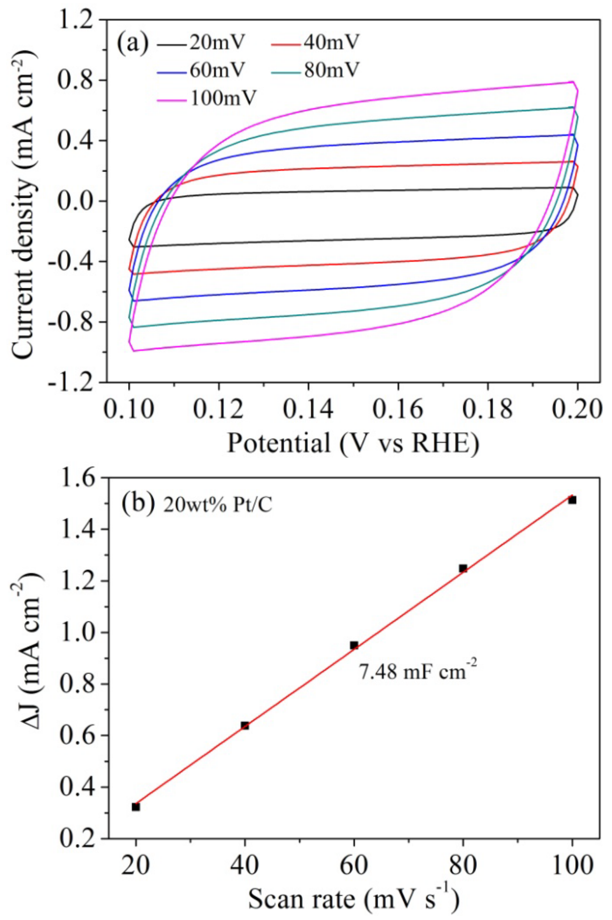

Supplementary Figure 15. (a) Cyclic voltammetry curves and (b) electrochemical double-layer capacitance of Pt/C supported on Ni plate.

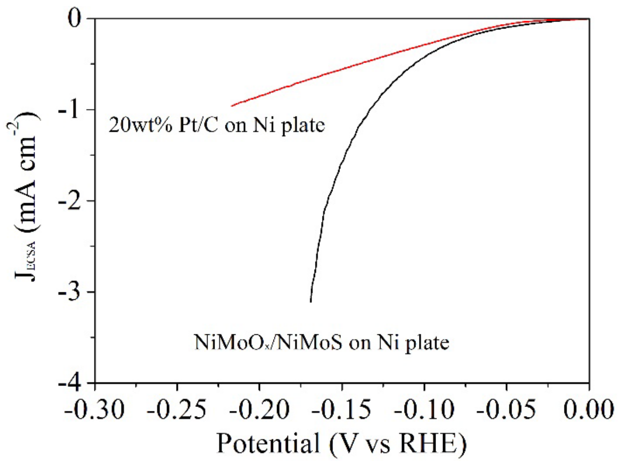

Supplementary Figure 16. Polarization curves of NiMoOx/NiMoS and commercial Pt/C supported on Ni plate with the relative current normalized to ECSA.

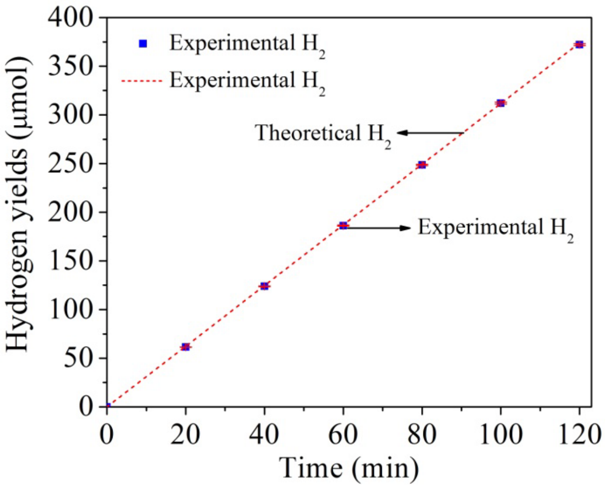

Supplementary Figure 17. The yields of hydrogen theoretically calculated from HER current and tested from gas chromatography by $NiMoO_x$/NiMoS at 10 mA cm^{-2}.

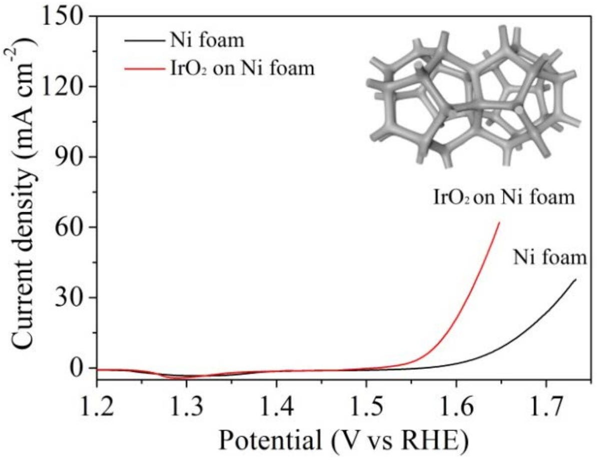

Supplementary Figure 18. OER polarization curves of commercial IrO_2 supported on Ni foam in comparison of pristine Ni foam in 1 M KOH.

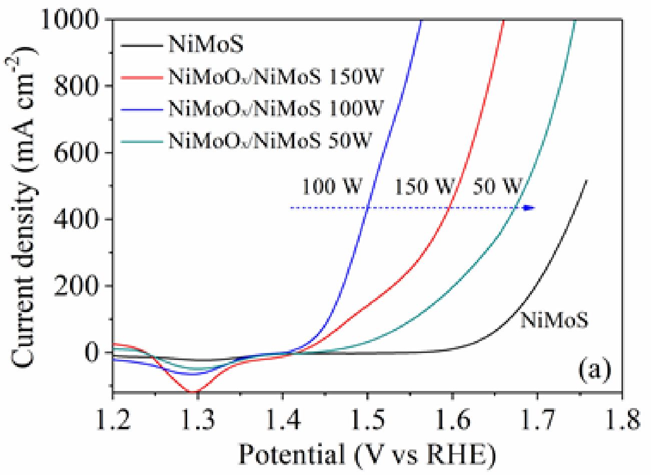

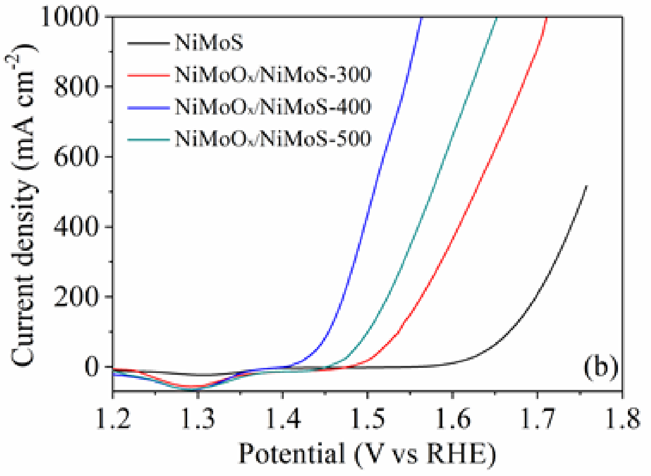

Supplementary Figure 19. LSV curves of different arrays for OER by (a) tunable power and (b) different hydrogenation temperatures.

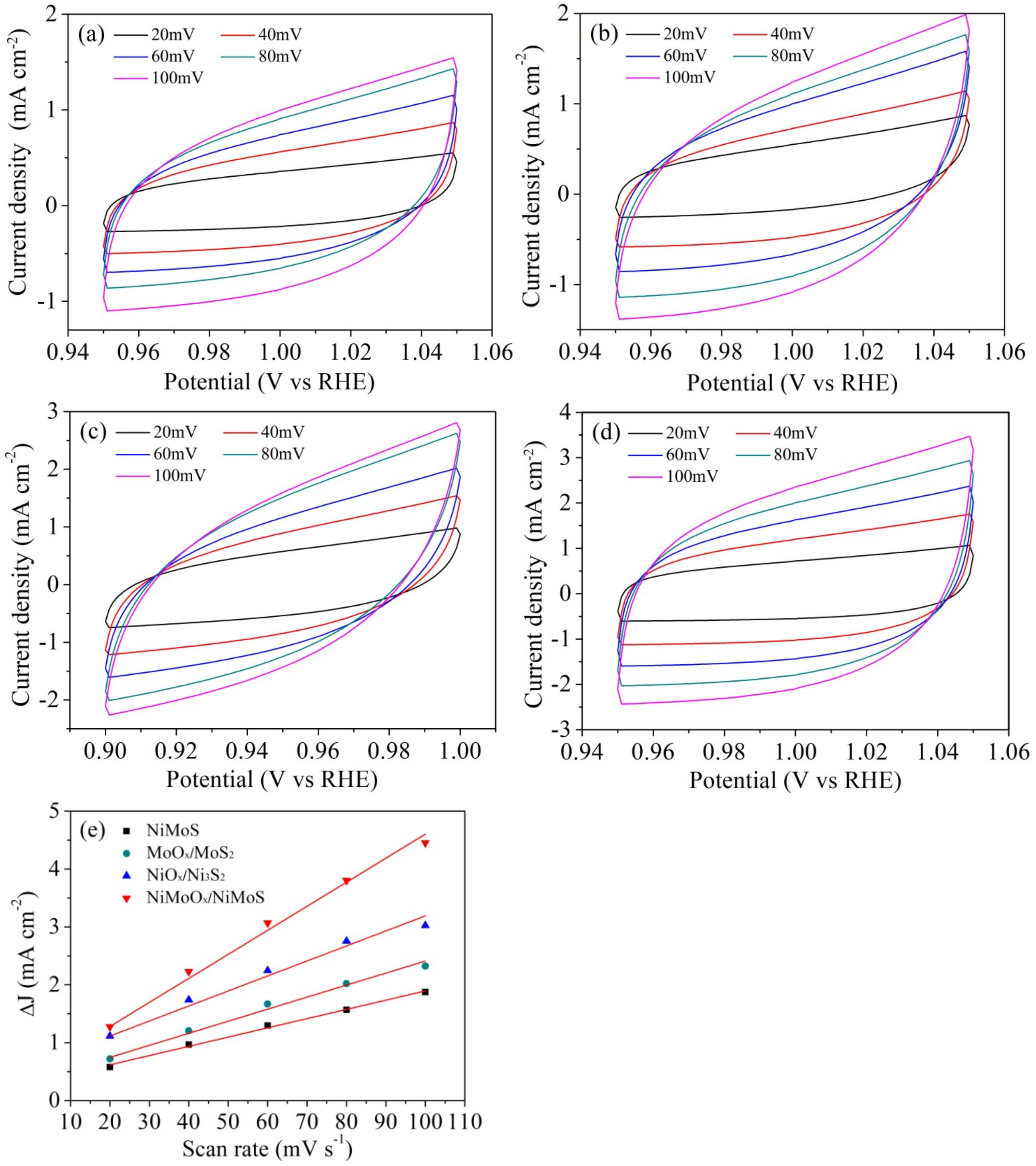

Supplementary Figure 20. (abcd) Cyclic voltammetry curves and (e) electrochemical double-layer capacitances of various catalysts supported on Ni foam during OER process.

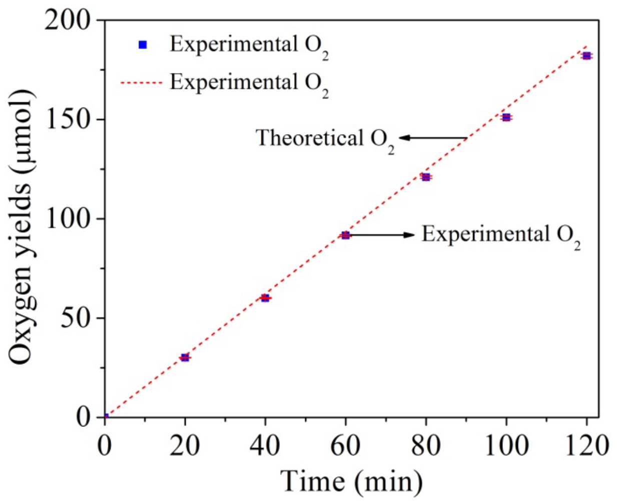

Supplementary Figure 21. The yields of hydrogen theoretically calculated from OER current and tested from gas chromatography by $NiMoO_x$/NiMoS at 10 mA cm^{-2}.

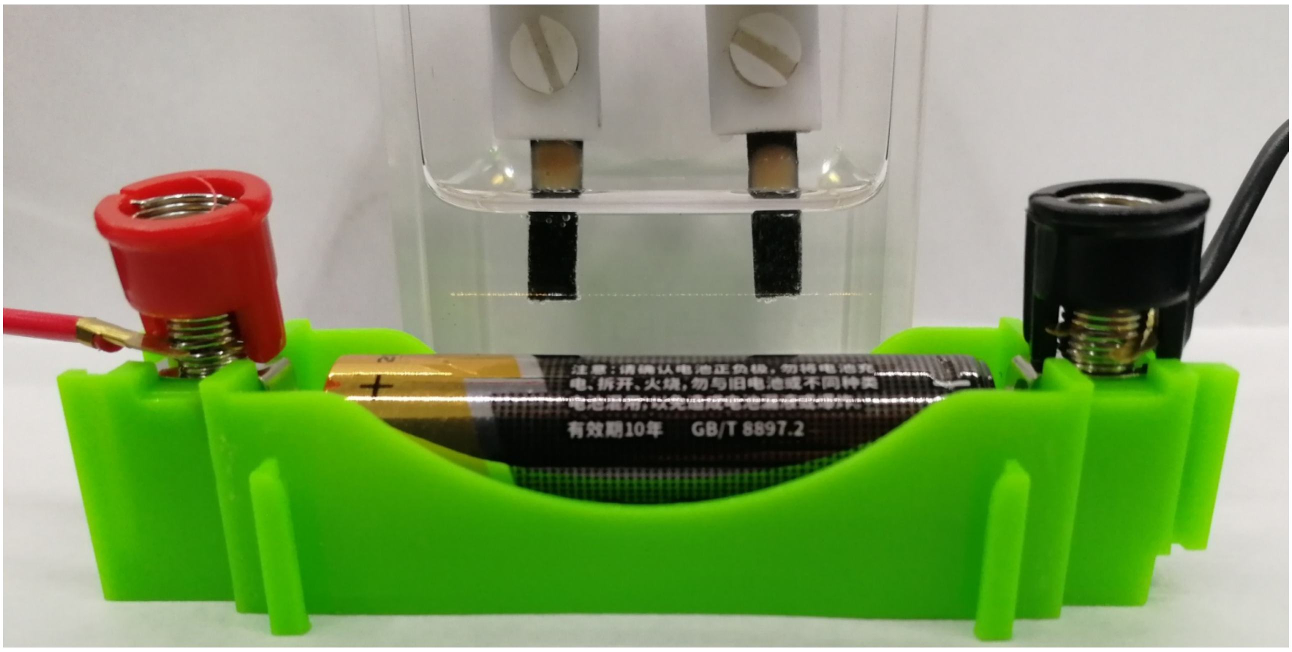

Supplementary Figure 22. H_2 and O_2 bubbles on its respective $NiMoO_x$/NiMoS heterostructure electrode for two-electrode overall water splitting by a 1.5 V AAA battery.

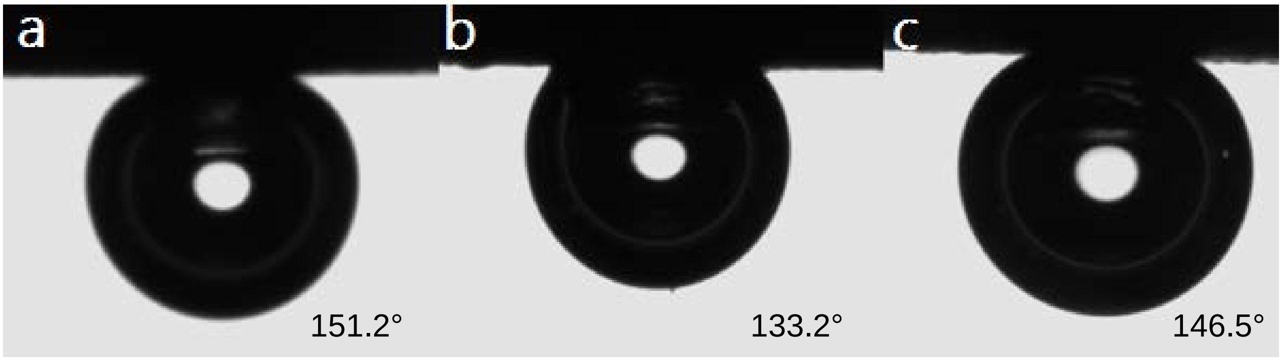

Supplementary Figure 23. Air-bubble contact angles under water for (a) $NiMoO_x$/NiMoS, (b) NiO_x/Ni_3S_2, (c) MoO_x/MoS_2.

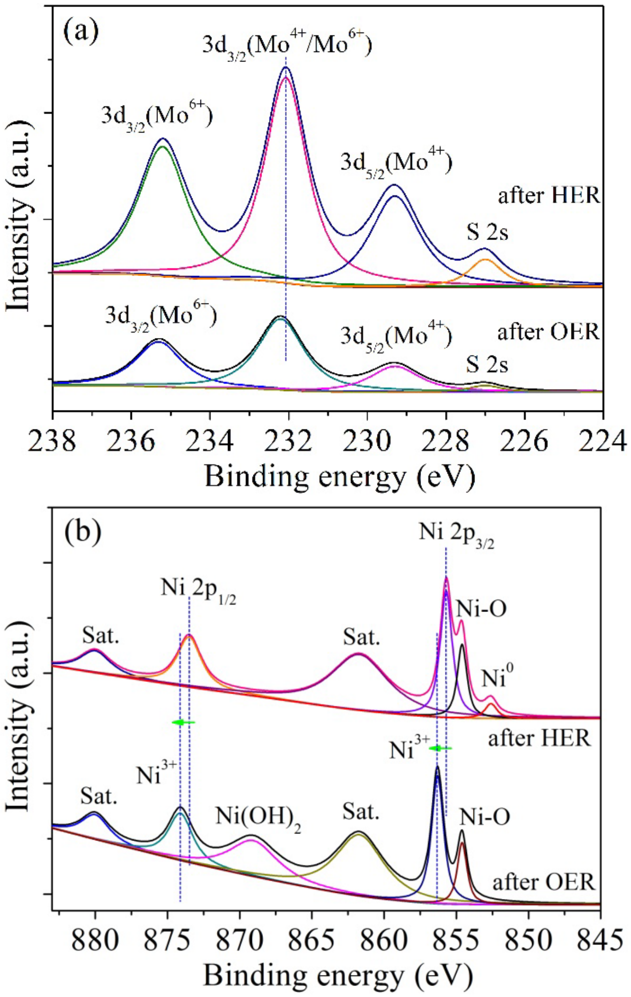

Supplementary Figure 24. XPS spectra of $NiMoO_x$/NiMoS array after HER and OER reactions.

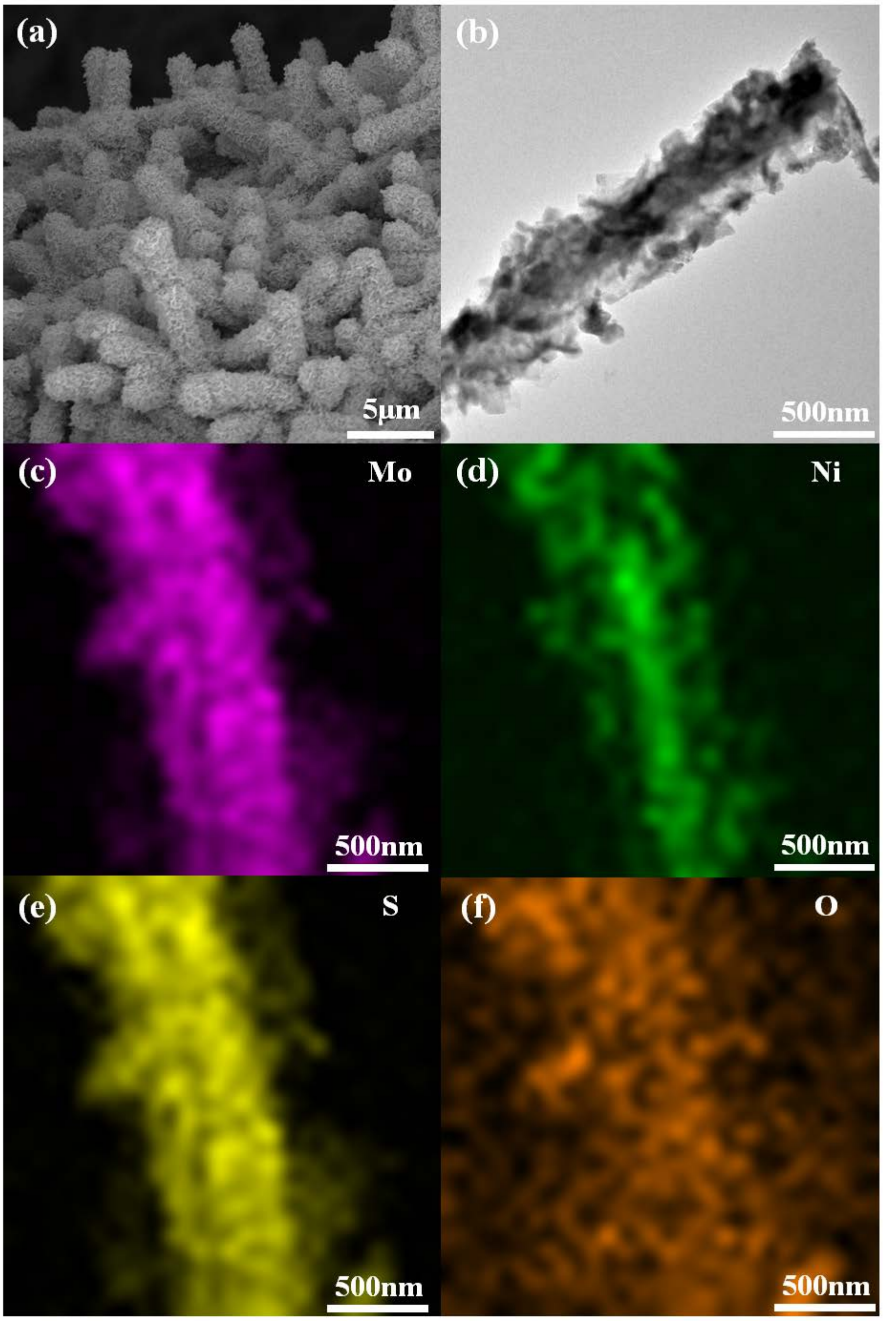

Supplementary Figure 25. SEM, TEM images and elements mapping of NiMoO$_x$/NiMoS array after electrocatalytic water splitting by two-electrode system.

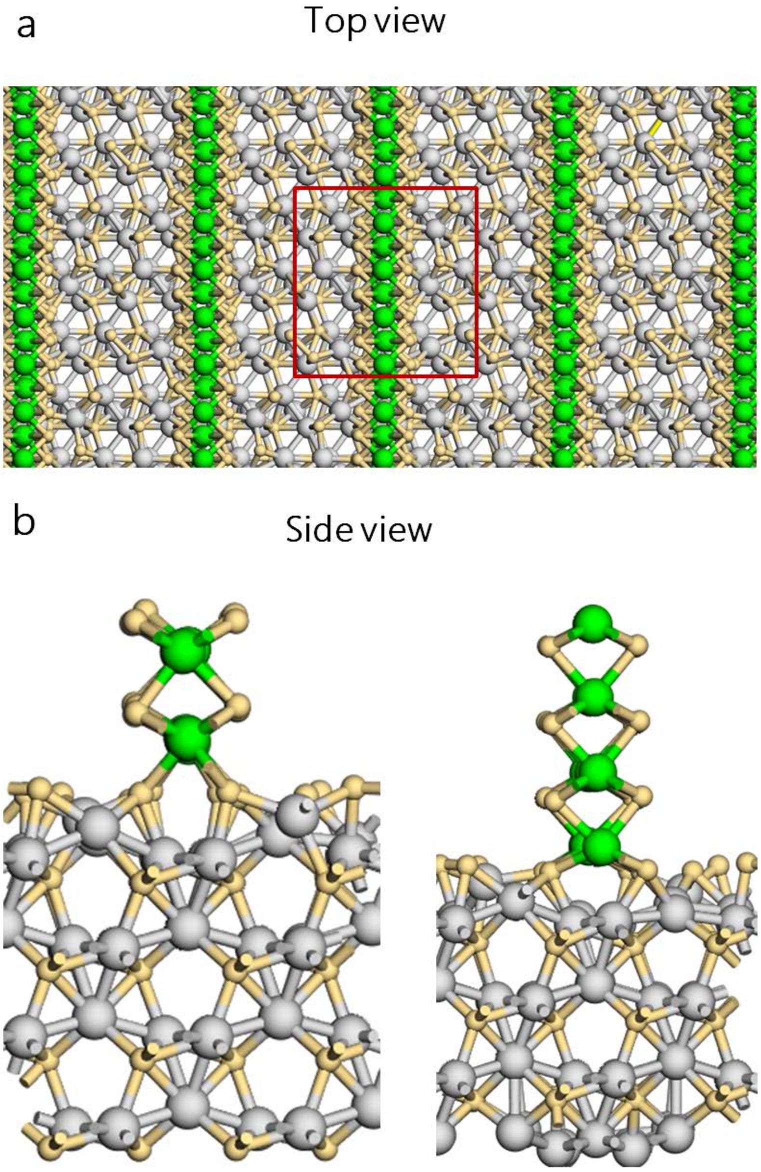

Supplementary Figure 26. (a) Top and (b) side view of DFT simulated Ni_3S_2/MoS_2 interface model. The unit cell is marked in the dark red boxes.

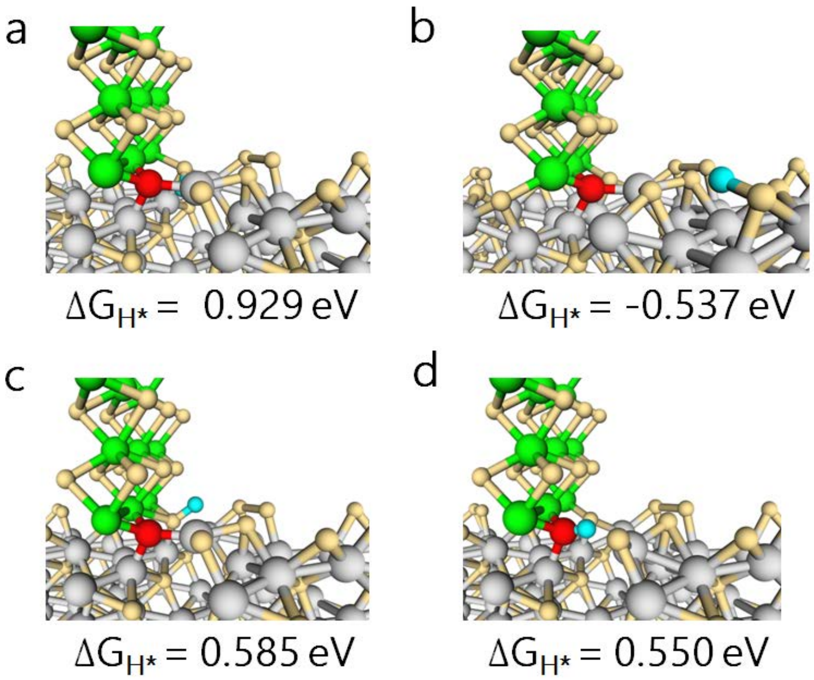

Supplementary Figure 27. Possible H* adsorption sites on the interface of $NiMoO_x$/NiMoS and corresponding free energies.

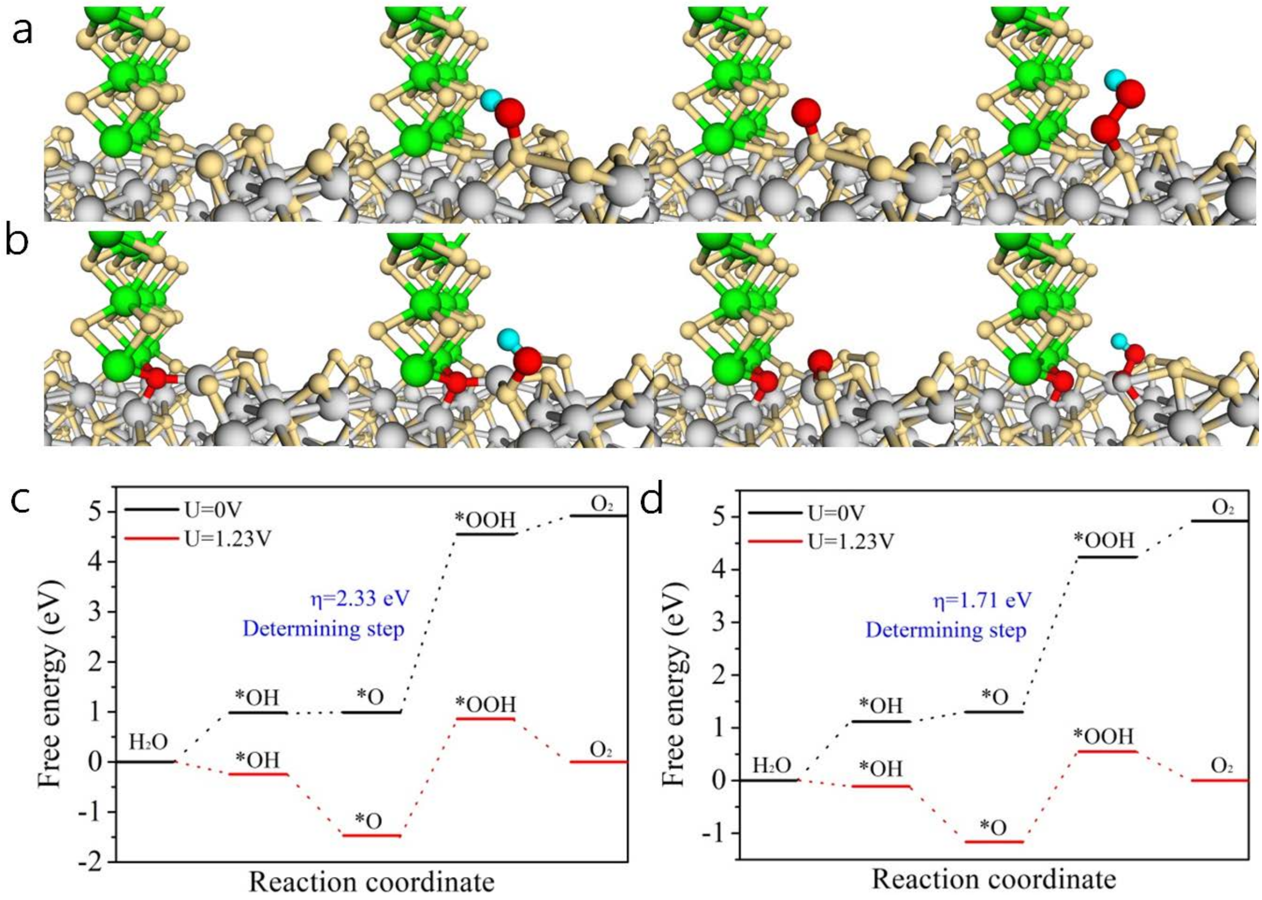

Supplementary Figure 28. (ab) OH, O and OOH adsorption configurations and (cd) free energy diagrams for OER on the surface of (ac) NiMoS and (bd) $NiMoO_x$/NiMoS.

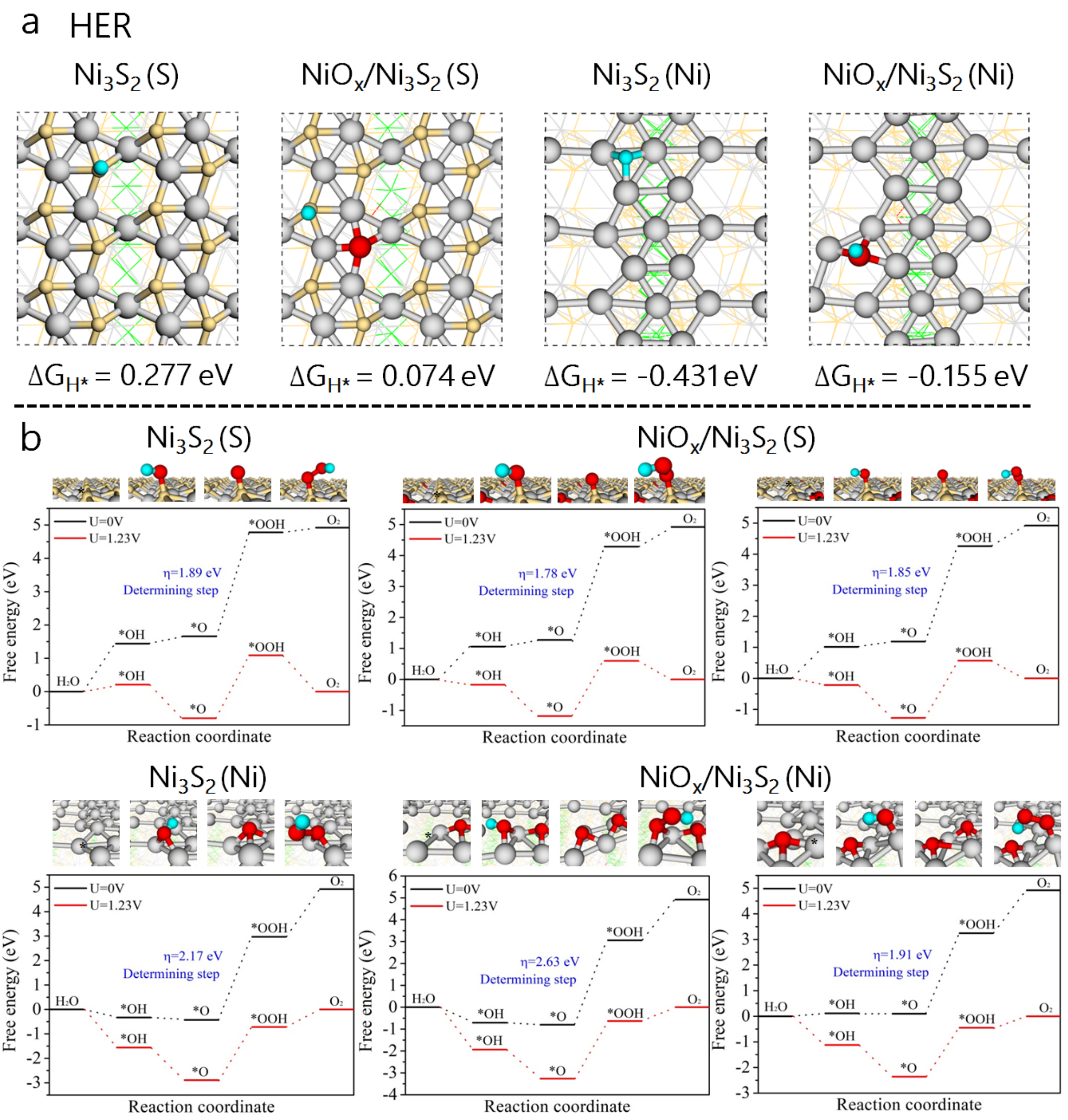

Supplementary Figure 29. (a) Chemisorption models and Gibbs free energies of H on the surface of Ni_3S_2 (S), NiO_x/Ni_3S_2 (S), Ni_3S_2(Ni) and NiO_x/Ni_3S_2 (Ni). (b) OH, O and OOH adsorption configurations for OER on the surface of Ni_3S_2 (S), Ni_3S_2 (Ni), NiO_x/Ni_3S_2 (S) and NiO_x/Ni_3S_2 (Ni) and free energy diagrams.

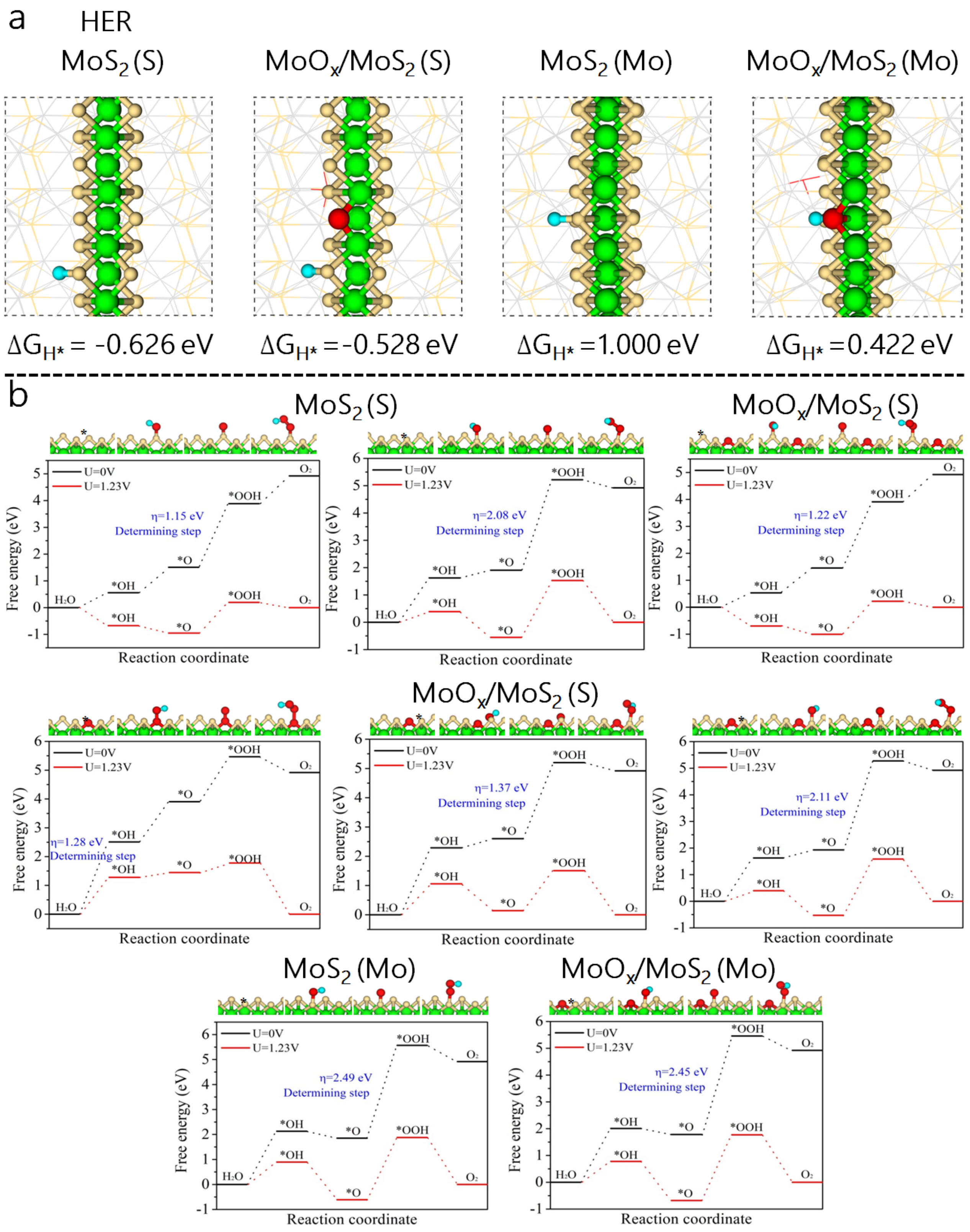

Supplementary Figure 30. (a) Chemisorption models and Gibbs free energies of H on the edge of MoS_2 (S), MoO_x/MoS_2 (S), MoS_2 (Mo) and MoO_x/MoS_2 (Mo). (b) OH, O and OOH adsorption configurations for OER on the edge of MoS_2 (S), MoO_x/MoS_2 (S), MoS_2 (Mo) and MoO_x/MoS_2 (Mo) and free energy diagrams.

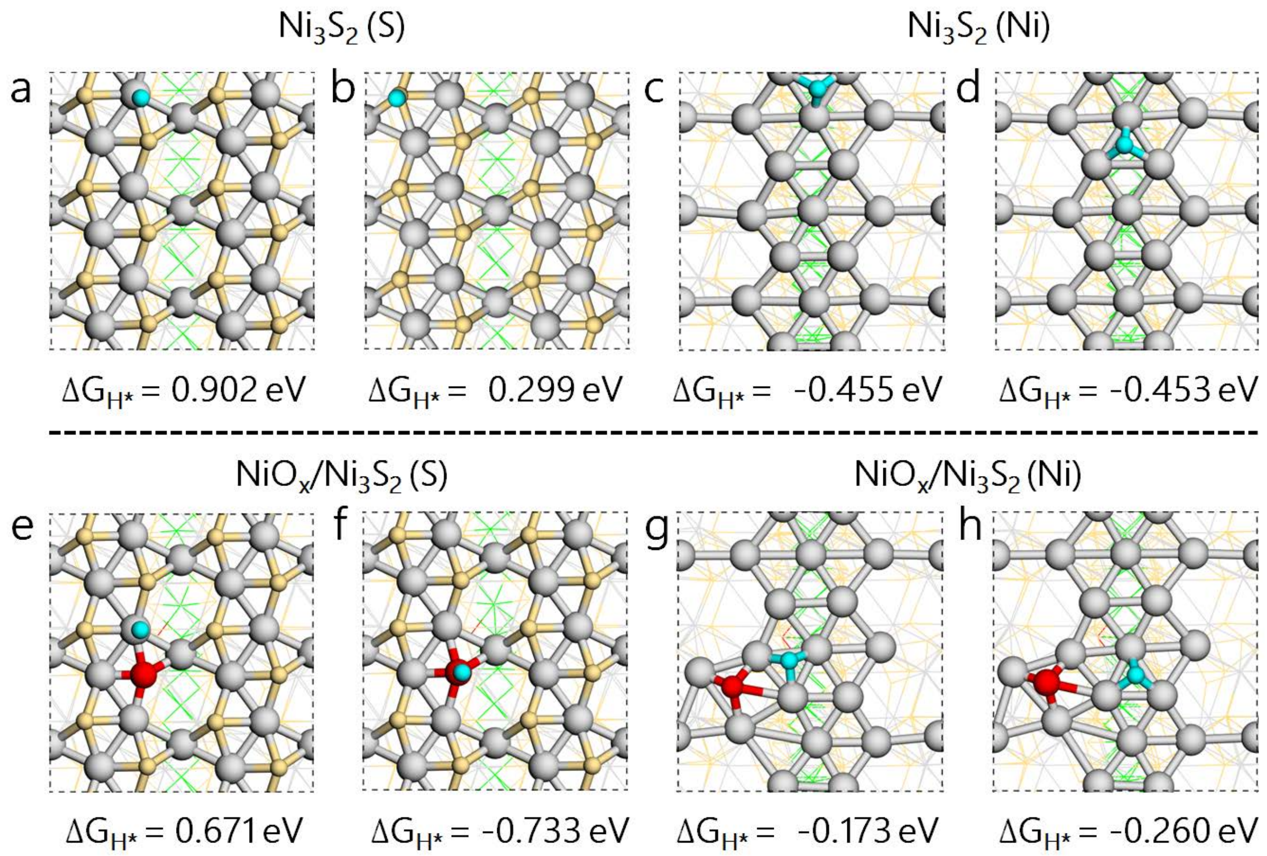

Supplementary Figure 31. Possible H adsorption sites on the surface of (a,b) Ni_3S_2 (S), (c,d) Ni_3S_2 (Ni), (e, f) NiO_x/Ni_3S_2 (S) and (g,h) NiO_x/Ni_3S_2 (Ni) and free energies.

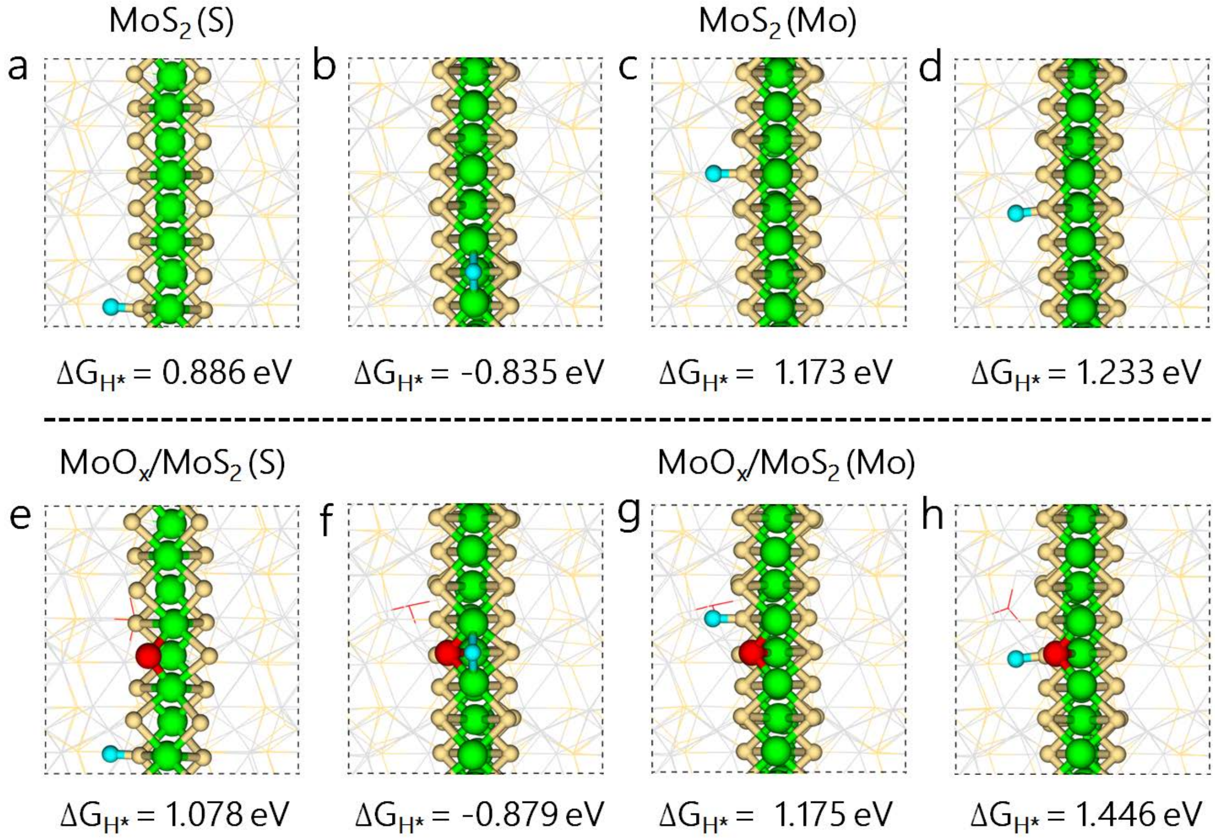

Supplementary Figure 32. Possible H adsorption sites on the edge of (a) MoS_2 (S), (b-d) MoS_2 (Mo), (e) MoO_x/MoS_2 (S) and (f-h) MoO_x/MoS_2 (Mo) and free energies.

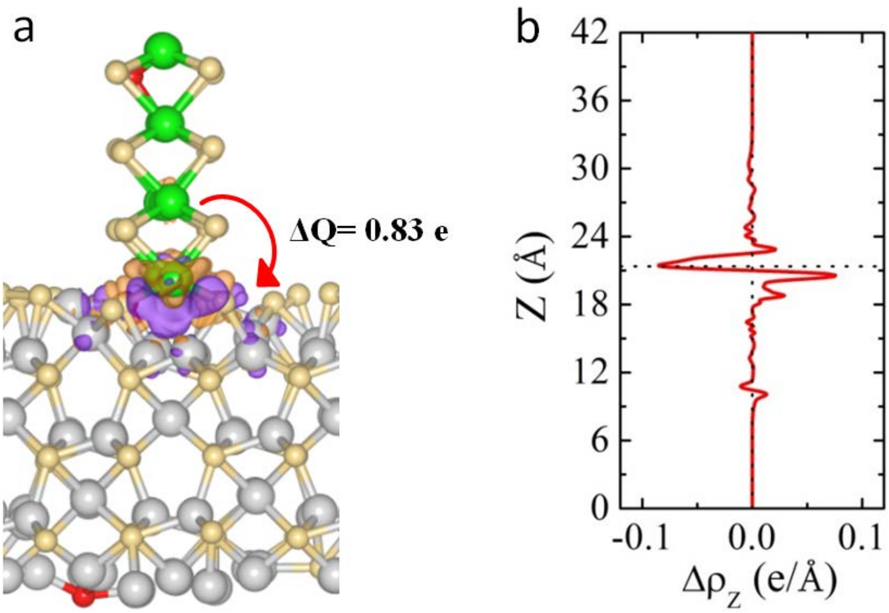

Supplementary Figure 33. (a) The charge density difference and (b) the plane-averaged charge density difference $\Delta\rho_Z$ along the Z direction of $NiMoO_x$/NiMoS. The purple and orange regions represent the charge accumulation and depletion, respectively.

Supplementary Table 1. Comparison of HER activities for various electrocatalysts.

Catalysts	Overpotential @10mA cm^{-2}	Tafel slope (mV/dec)	Reference
Pt_1/N-C	46 mV	36.8	[1]
P,W co doped Co_3N	41 mV	40	[2]
C-doped MoS_2	45 mV	46	[3]
FeCoNi-HNTAs	58 mV	37.5	[4]
Ni-Co with 1T MoS_2	70 mV	38.1	[5]
$MoS_2/Co_9S_8/Ni_3S_2$/Ni	113 mV	85	[6]
(Ru-Co)O_x	44.1 mV	23.5	[7]
sc-$Ni_2P^{\delta-}$/NiHO	60 mV	75	[8]
Ni-MoS_2	98 mV	60	[9]
MoO_3/Ni-NiO	62 mV	59	[10]
Co-Ni_3N	194 mV	156	[11]
R-MoS_2@NF	71 mV	100	[12]
Co@N-CNTs@rGO	108 mV	55	[13]
O-CoMoS	97 mV	70	[14]
$Ni(OH)_2/MoS_2$	80 mV	60	[15]
MoS_2/NiS_2	62 mV	50.1	[16]
MoS_2/NiS NCs	92 mV	113	[17]
$NiMoO_x$/NiMoS	38 mV	38	This work

Supplementary Table 2. The turnover frequency (TOF) per site for various HER electrocatalysts at different overpotentials in alkaline condition.

Catalysts	TOF values		
	η=50 mV	η=100 mV	η=200 mV
$NiMoO_x$/NiMoS	0.28 s^{-1}	1.97 s^{-1}	10.08 s^{-1}
NiO_x/Ni_3S_2	0.16 s^{-1}	0.59 s^{-1}	3.80 s^{-1}
MoO_x/MoS_2	0.007 s^{-1}	0.027 s^{-1}	0.35 s^{-1}
NiMoS	0.004 s^{-1}	0.0435 s^{-1}	0.13 s^{-1}

Supplementary Table 3. Comparison of TOF values of HER catalysts in alkaline condition.

Catalysts	TOF (H_2 s^{-1}@ mV)	Reference
$NiCo_2P_x$	0.056 s^{-1} at 100 mV	18
$MoNi_4/MoO_{3-x}$	1.13 s^{-1} at 100 mV	19
N-$NiCo_2S_4$	1.0 s^{-1} at 125 mV	20
NiO@1T MoS_2	0.7 s^{-1} at 130 mV	21
$Mo_1N_1C_2$	1.46 s^{-1} at 150 mV	22
Co-NiS_2	4.1 s^{-1} at 200 mV	23
MoO_3@MoS_2	1.93 s^{-1} at 250 mV	24
$NiMoO_x$/NiMoS	0.28 s^{-1} at 50 mV	this work
$NiMoO_x$/NiMoS	1.97 s^{-1} at 100 mV	this work

Supplementary Table 4. Comparison of mass activity of HER electrocatalysts.

Catalysts	Mass activity (A g^{-1})	Reference
Ru@MWCNT	186 A g^{-1} at 20mV	[25]
Co-NiS_2	100 A g^{-1} at 185mV	[23]
NiS_2	150 A g^{-1} at 300mV	[23]
NiCoN/C	204 A g^{-1} at 200mV	[26]
R-MoS_2@NF	40 A g^{-1} at 100mV	[12]
A-MoS_2	40 A g^{-1} at 300mV	[12]
Pt-CoS_2/CC	2.1 A g^{-1} at 100mV	[27]
Pt@PCM	600 A g^{-1} at 185mV	[28]
O_V-Co_3O_4	2.12 A g^{-1} at 200mV	[29]
$NiMoO_x$/NiMoS	436 A g^{-1} at 200mV	this work

Supplementary Table 5. Comparison of OER activities for various electrocatalysts.

Catalysts	Overpotential @10mA cm^{-2}	Tafel slope (mV/dec)	Reference
np-Ir/NiFeO	197 mV	29.6	[30]
CoBDC-$Fc_{0.17}$	291 mV	61	[31]
W-$Ni(OH)_2$	237 mV	33	[32]
Ru/CoFe-LDHs	198 mV	39	[33]
Ni-Co with 1T MoS_2	235 mV	45.7	[5]
Ir-NiO	215 mV	38	[34]
FeOOH(Se)/IF	287 mV	54	[35]
$MoS_2/Co_9S_8/Ni_3S_2$/Ni	166 mV	58	[6]
P-Co_3O_4	280 mV	51.6	[36]
Ni/$Ni(OH)_2$	270 mV	53	[37]
W_2N/WC	320 mV	122.8	[38]
NiTe/NiS	244 mV	49	[39]
$Co_{1.8}Ni(OH)_{5.6}$ @$Co_{1.8}NiS_{0.4}(OH)_{4.8}$	274 mV	45	[40]
δ-FeOOH NSs/NF	265 mV	69	[41]
Co-Ni_3N	307 mV	57	[11]
Porous Ni_3S_4	257 mV	67	[42]
O-CoMoS	272 mV	45	[14]
$NiMoO_x$/NiMoS	186 mV	34	This work

Supplementary Table 6. Comparison of overall water splitting activities of bifunctional electrocatalysts.

Catalysts	Electrolyte	Potential	Reference
$RuIrO_x$	alkaline	1.47V	[43]
a-RuTe	acid	1.52V	[44]
Ni-Fe NPs	alkaline	1.47V	[45]
IFONFs	alkaline	1.58V	[46]
NiFe-NiMo/Ni-P	alkaline	1.51V	[47]
NiFe MOF	alkaline	1.55V	[48]
$NiFeO_x/CNF$	alkaline	1.51V	[49]
CoMoNiS-NF	alkaline	1.54V	[6]
EBP@EG	alkaline	1.54V	[50]
$R\text{-}NiCo_2O_4$	alkaline	1.61V	[51]
CoP/NCNHP	alkaline	1.64V	[52]
Ni_3S_2	alkaline	1.76V	[53]
CoMnO@CN	alkaline	1.5V	[54]
$MoO_3/Ni\text{-}NiO$	alkaline	1.55V	[10]
$Ni/Ni(OH)_2$	alkaline	1.59V	[37]
$Co_2P/CoNPC$	alkaline	1.64V	[55]
W_2N/WC	alkaline	1.58V	[38]
CoFeZr oxides	alkaline	1.63V	[56]
Cr-FeNi-P/NCN	alkaline	1.50V	[57]
Co/CNFs	alkaline	1.69V	[58]

Co_3S_4/EC-MOF	alkaline	1.55V	[59]
Co-RuIr	acid	1.52V	[60]
$Fe_{0.09}Co_{0.13}$-$NiSe_2$	alkaline	1.52V	[61]
Se-(NiCo)S/OH	alkaline	1.6V	[62]
$SrCo_{0.85}Fe_{0.1}P_{0.05}O_{3-\delta}$	alkaline	1.66V	[63]
Co-MoS_2/BCCF	alkaline	1.55V	[64]
CoP NC	alkaline	1.56V	[65]
δ-FeOOH NSs/NF	alkaline	1.62V	[41]
Pt/$LiCoO_2$	alkaline	1.54V	[66]
Co/β-Mo_2C@N-CNTs	alkaliine	1.64V	[67]
Ir_1@Co/NC	alkaline	1.603V	[68]
RuCu NSs/C	alkaline	1.49V	[69]
$CoSn_2$	alkaline	1.55V	[70]
Fe-Ni@NC-CNTs	alkaline	1.58V	[71]
$Ni_{0.1}Co_{0.9}P$	neutral	1.89V	[72]
Ni_5P_4	alkaline	1.70 V	[73]
Ni/Mo_2C	alkaline	1.64 V	[74]
Pt-CoS_2/CC	alkaline	1.55 V	[27]
Fe-Ni-MoN	alkaline	1.51 V	[75]
N-$NiMoO_4$/NiS_2	alkaline	1.6 V	[76]
NC/CuCo/$CuCoO_x$	alkaline	1.53 V	[77]
NC/NiCu/NiCuN	alkaline	1.56V	[78]

$NiCo_2S_4$	alkaline	1.68 V	[79]
O-CoMoS	alkaline	1.60 V	[14]
P-Co_3O_4/NF	alkaline	1.63 V	[80]
$NiMoO_x$/NiMoS	alkaline	1.46V	this work

Supplementary Table 7. Comparison of overall water splitting activities of bifunctional electrocatalysts with high current at 500 mA cm^{-2}.

Catalysts	Potential	Reference
FeP/Ni_2P	1.72 V	[81]
RFNOH-10‖NiFe-LDH/NF	1.69 V	[82]
Cu@NiFe LDH‖$Ni_{2(1-x)}Mo_{2x}P$	1.82 V	[83]
Ni NWs‖$Ni_{0.8}Fe_{0.2}$-AHNA	1.702 V	[84]
$MoNi_4$/SSW‖SSW Rs-12h	1.978 V	[85]
NFN-MOF/NF	1.9 V	[86]
$NiMoO_x$/NiMoS‖$NiMoO_x$/NiMoS	1.6 V	this work

Supplementary Table 8. Entropy and zero point energy used for calculated the free energy of the intermediate in this work.

	TS (eV)	TΔS (eV)	ZPE (eV)	ΔZPE (eV)	ΔZPE-TΔS(eV)
H_2O[1]	0.67	0	0.56	0	0
*OH+1/2H_2	0.33	-0.34	0.50	-0.06	0.28
*O+H_2	0.48	-0.19	0.34	-0.22	-0.03
*OOH+3/2H_2	0.75	-0.59	0.87	-0.25	0.34
H_2[87,88]	0.41	-	0.27	-	-
*OH	0.12	-	0.36	-	-
*O	0.07	-	0.07	-	-
*OOH	0.13	-	0.46	-	-

Supplementary Note 1. Calculation of TOF.

The TOF values (s^{-1}) were calculated with the following formula:

$$\text{TOF}=\frac{I}{2nF} \qquad (1)$$

I: current density extracted from the LSV curves;

F: Faraday constant;

n: the number of active sites. Cycle voltammetry measurements were conducted between -0.2 V and 0.6 V *vs.* RHE in 1M PBS (pH=7) at a scan rate of 50 mV s^{-1}. The absolute components of the voltammetric charges tested during one CV cycle were calculated. Assuming a one-electron process for both reduction and oxidation, the absolute charges was divided by two and the Faraday constant to get the number of active sites of the catalysts. The upper limit of active sites for $NiMoO_x$/NiMoS could be calculated according to the equation:

$$n=Q/2F \qquad (2)$$

Supplementary References

1. Fang, S. et al. Uncovering near-free platinum single-atom dynamics during electrochemical hydrogen evolution reaction. *Nat. Commun.* **11**, 1029 (2020).
2. Liu, Y. et al. Manipulating dehydrogenation kinetics through dual-doping Co_3N electrode enables highly efficient hydrazine oxidation assisting self-powered H_2 production. *Nat. Commun.* **11**, 1853 (2020).
3. Zang, Y. et al. Tuning orbital orientation endows molybdenum disulfide with exceptional alkaline hydrogen evolution capability. *Nat. Commun.* **10**, 1217 (2019).
4. Li, H. et al. Systematic design of superaerophobic nanotube-array electrode comprised of transition-metal sulfides for overall water splitting. *Nat. Commun.* **9**, 2452 (2018).
5. Li, H. et al. Amorphous nickel-cobalt complexes hybridized with 1T-phase molybdenum disulfide via hydrazine-induced phase transformation for water splitting. *Nat. Commun.* **8**, 15377 (2017).
6. Yang, Y. et al. Hierarchical nanoassembly of $MoS_2/Co_9S_8/Ni_3S_2/Ni$ as a highly efficient electrocatalyst for overall water splitting in a wide pH range. *J. Am. Chem. Soc.* **141**, 10417-10430 (2019).
7. Wang, C. and Qi, L. Heterostructured inter-doped ruthenium-cobalt oxide hollow nanosheet arrays for highly efficient overall water splitting. *Angew. Chem. Int. Ed.* **59,** 17219-17224 (2020).
8. You, B. et al. Negative charging of transition-metal phosphides via strong electronic coupling for destabilization of alkaline water. *Angew. Chem. Int. Ed.* **58**, 11796-11800 (2019).
9. Zhang, J. et al. Engineering water dissociation sites in MoS_2 nanosheets for accelerated electrocatalytic hydrogen production. *Energy Environ. Sci.* **9**, 2789-2793 (2016).
10. Li, X. et al. Sequential electrodeposition of bifunctional catalytically active structures in MoO_3/Ni-NiO composite electrocatalysts for selective hydrogen and oxygen evolution. *Adv. Mater.* **32,** 2003414 (2020).
11. Zhu, C. et al. In situ grown epitaxial heterojunction exhibits high-performance electrocatalytic water splitting. *Adv. Mater.* **30**, 1705516 (2018).
12. Anjum, M. A. R. et al. Efficient hydrogen evolution reaction catalysis in alkaline media by all-in-one MoS_2 with multifunctional active sites. *Adv. Mater.* **30**, 1707105 (2018).
13. Chen, Z. et al. Ultrafine Co nanoparticles encapsulated in carbon-nanotubes-grafted graphene sheets as advanced electrocatalysts for the hydrogen evolution reaction. *Adv. Mater.* **30**, 1802011 (2018).
14. Hou, J. et al. Vertically aligned oxygenated-CoS_2-MoS_2 heteronanosheet architecture from polyoxometalate for efficient and stable overall water splitting. *ACS Catal.* **8**, 4612-4621 (2018).
15. Zhang, B. et al. Interface engineering: The $Ni(OH)_2/MoS_2$ heterostructure for highly efficient alkaline hydrogen evolution. *Nano Energy* **37**, 74-80 (2017).
16. Lin, J. et al. Defect-rich heterogeneous MoS_2/NiS_2 nanosheets electrocatalysts for efficient overall water splitting. *Adv. Sci.* **6**, 1900246 (2019).

17. Zhai, Z. et al. Dimensional construction and morphological tuning of heterogeneous MoS_2/NiS electrocatalysts for efficient overall water splitting. *J. Mater. Chem. A* **6**, 9833-9838 (2018).
18. Zhang, R. et al. Ternary $NiCo_2P_x$ nanowires as pH-universal electrocatalysts for highly efficient hydrogen evolution reaction. *Adv. Mater.* **29**, 1605502 (2017).
19. Chen, Y. Y. et al. Self-templated fabrication of $MoNi_4/MoO_{3-x}$ nanorod arrays with dual active components for highly efficient hydrogen evolution. *Adv. Mater.* **29**, 1703311 (2017).
20. Wu, Y. et al. Electron density modulation of $NiCo_2S_4$ nanowires by nitrogen incorporation for highly efficient hydrogen evolution catalysis. *Nat. Commun.* **9**, 1425 (2018).
21. Huang, Y. et al. Atomically engineering activation sites onto metallic 1T-MoS_2 catalysts for enhanced electrochemical hydrogen evolution. *Nat. Commun.* **10**, 982 (2019).
22. Chen, W. et al. Rational design of single molybdenum atoms anchored on n-doped carbon for effective hydrogen evolution reaction. *Angew. Chem. Int. Ed.* **56**, 16086-16090 (2017).
23. Yin, J. et al. Atomic arrangement in metal-doped NiS_2 boosts the hydrogen evolution reaction in alkaline media. *Angew. Chem. Int. Ed.* **58**, 18676-18682 (2019).
24. Huang, L. B. et al. Self-limited on-site conversion of MoO_3 nanodots into vertically aligned ultrasmall monolayer MoS_2 for efficient hydrogen evolution. *Adv. Energy Mater.* **8**, 1800734 (2018).
25. Kweon, D. H. et al. Ruthenium anchored on carbon nanotube electrocatalyst for hydrogen production with enhanced Faradaic efficiency. *Nat. Commun.* **11**, 1278 (2020).
26. Lai, J. et al. Strongly coupled nickel-cobalt nitrides/carbon hybrid nanocages with Pt-like activity for hydrogen evolution catalysis. *Adv. Mater.* **31**, 1805541 (2019).
27. Han, X. et al. Ultrafine Pt nanoparticle−decorated pyrite−type CoS_2 nanosheet arrays coated on carbon cloth as a bifunctional electrode for overall water splitting. *Adv. Energy Mater.* **8**, 1800935 (2018).
28. Zhang, H. B. et al. Dynamic traction of lattice-confined platinum atoms into mesoporous carbon matrix for hydrogen evolution reaction. *Sci. Adv.* **4,** eaao6657 (2018).
29. Zhang, H. et al. Continuous oxygen vacancy engineering of the Co_3O_4 layer for an enhanced alkaline electrocatalytic hydrogen evolution reaction. *J. Mater. Chem. A* **7**, 13506-13510 (2019).
30. Jiang, K. et al. Dynamic active-site generation of atomic iridium stabilized on nanoporous metal phosphides for water oxidation. *Nat. Commun.* **11**, 2701 (2020).
31. Xue, Z. et al. Missing-linker metal-organic frameworks for oxygen evolution reaction. *Nat. Commun.* **10**, 5048 (2019).
32. Yan, J. et al. Single atom tungsten doped ultrathin α-$Ni(OH)_2$ for enhanced electrocatalytic water oxidation. *Nat. Commun.* **10**, 2149 (2019).
33. Li, P. et al. Boosting oxygen evolution of single-atomic ruthenium through electronic coupling with cobalt-iron layered double hydroxides. *Nat. Commun.* **10**, 1711 (2019).

34. Wang, Q. et al. Ultrahigh-loading of Ir single atoms on NiO matrix to dramatically enhance oxygen evolution reaction. *J. Am. Chem. Soc.* **142**, 7425-7433 (2020)
35. Niu, S. et al. Se-doping activates FeOOH for cost-effective and efficient electrochemical water oxidation. *J. Am. Chem. Soc.* **141**, 7005-7013 (2019).
36. Xiao, Z. et al. Filling the oxygen vacancies in Co_3O_4 with phosphorus: an ultra-efficient electrocatalyst for overall water splitting. *Energy Environ. Sci.* **10**, 2563-2569 (2017).
37. Dai, L. et al. Ultrathin Ni(0)-embedded $Ni(OH)_2$ heterostructured nanosheets with enhanced electrochemical overall water splitting. *Adv. Mater.* **32**, 1906915 (2020).
38. Diao, J. et al. Interfacial engineering of W_2N/WC heterostructures derived from solid-state synthesis: a highly efficient trifunctional electrocatalyst for ORR, OER, and HER. *Adv. Mater.* **32**, 1905679 (2020).
39. Xue, Z. et al. Interfacial electronic structure modulation of NiTe nanoarrays with NiS nanodots facilitates electrocatalytic oxygen evolution. *Adv. Mater.* **31**, 1900430 (2019).
40. Wang, B. et al. A nanosized CoNi hydroxide@hydroxysulfide core-shell heterostructure for enhanced oxygen evolution. *Adv. Mater.* **31**, 1805658 (2019).
41. Liu, B. et al. Iron vacancies induced bifunctionality in ultrathin feroxyhyte nanosheets for overall water splitting. *Adv. Mater*. **30** 1803144 (2018).
42. Wan, K. et al. Hierarchical porous Ni_3S_4 with enriched high-valence Ni sites as a robust electrocatalyst for efficient oxygen evolution reaction. *Adv. Funct. Mater.* **29**, 1900315 (2019).
43. Zhuang, Z. et al. Three-dimensional open nano-netcage electrocatalysts for efficient pH-universal overall water splitting. *Nat. Commun.* **10**, 4875 (2019).
44. Wang, J. et al. Amorphization activated ruthenium-tellurium nanorods for efficient water splitting. *Nat. Commun.* **10**, 5692 (2019).
45. Suryanto, B. H. R. et al. Overall electrochemical splitting of water at the heterogeneous interface of nickel and iron oxide. *Nat. Commun.* **10**, 5599 (2019).
46. Fan, X. et al. Defect-enriched iron fluoride-oxide nanoporous thin films bifunctional catalyst for water splitting. *Nat. Commun.* **9**, 1809 (2018).
47. Sahasrabudhe, A. et al. Value added transformation of ubiquitous substrates into highly efficient and flexible electrodes for water splitting. *Nat. Commun.* **9**, 2014 (2018).
48. Duan, J. et al. Ultrathin metal-organic framework array for efficient electrocatalytic water splitting. *Nat. Commun.* **8**, 15341 (2017).
49. Wang, H. et al. Bifunctional non-noble metal oxide nanoparticle electrocatalysts through lithium-induced conversion for overall water splitting. *Nat. Commun.* **6**, 7261 (2015).
50. Yuan, Z. et al. Ultrathin black phosphorus-on-nitrogen doped graphene for efficient overall water splitting: dual modulation roles of directional interfacial charge transfer. *J. Am. Chem. Soc.* **141**, 4972-4979 (2019).
51. Peng, S. et al. Necklace-like multishelled hollow spinel oxides with oxygen vacancies for efficient water electrolysis. *J. Am. Chem. Soc.* **140**, 13644-13653 (2018).

52. Pan, Y. et al. Core-shell ZIF-8@ZIF-67-derived CoP nanoparticle-embedded N-doped carbon nanotube hollow polyhedron for efficient overall water splitting. *J. Am. Chem. Soc.* **140**, 2610-2618 (2018).
53. Feng, L. L. et al. High-index faceted Ni_3S_2 nanosheet arrays as highly active and ultrastable electrocatalysts for water splitting. *J. Am. Chem. Soc.* **137**, 14023-14026 (2015).
54. Li, J. et al. Nanoparticle superlattices as efficient bifunctional electrocatalysts for water splitting. *J. Am. Chem. Soc.* **137**, 14305-14312 (2015).
55. Liu, H. et al. Metal-organic framework-derived Co_2P nanoparticle/multi-doped porous carbon as a trifunctional electrocatalyst. *Adv. Mater*. **32,** 2003649 (2020).
56. Huang, L. et al. Zirconium-regulation-induced bifunctionality in 3D cobalt-iron oxide nanosheets for overall water splitting. *Adv. Mater.* **31**, 1901439 (2019).
57. Wu, Y. et al. Cr-doped FeNi-P nanoparticles encapsulated into N-doped carbon nanotube as a robust bifunctional catalyst for efficient overall water splitting. *Adv. Mater.* **31**, 1900178 (2019).
58. Yang, Z. et al. Trifunctional self-supporting cobalt-embedded carbon nanotube films for ORR, OER, and HER triggered by solid diffusion from bulk metal. *Adv. Mater.* **31**, 1808043 (2019).
59. Liu, T. et al. Self-sacrificial template-directed vapor-phase growth of mof assemblies and surface vulcanization for efficient water splitting. *Adv Mater* **31**, 1806672 (2019).
60. Shan, J. et al. Transition-metal-doped RuIr bifunctional nanocrystals for overall water splitting in acidic environments. *Adv. Mater.* **31**, 1900510 (2019).
61. Sun, Y. et al. Strong electronic interaction in dual-cation-incorporated $nise_2$ nanosheets with lattice distortion for highly efficient overall water splitting. *Adv. Mater.* **30**, 1802121 (2018).
62. Hu, C. et al. Synergism of geometric construction and electronic regulation: 3D Se-$(NiCo)S_x/(OH)_x$ nanosheets for highly efficient overall water splitting. *Adv. Mater.* **30**, 1705538 (2018).
63. Chen, G. et al. A universal strategy to design superior water-splitting electrocatalysts based on fast in situ reconstruction of amorphous nanofilm precursors. *Adv. Mater.* **30**, 1804333 (2018).
64. Xiong, Q. et al. Cobalt covalent doping in MoS_2 to induce bifunctionality of overall water splitting. *Adv. Mater*. **30**, 1801450 (2018).
65. Li, H. et al. Colloidal cobalt phosphide nanocrystals as trifunctional electrocatalysts for overall water splitting powered by a zinc-air battery. *Adv. Mater.* **30**, 1705796 (2018).
66. Zheng, X. et al. Multifunctional active-center-transferable platinum/lithium cobalt oxide heterostructured electrocatalysts towards superior water splitting. *Angew. Chem. Int. Ed.* **59,** 14533-14540 (2020).
67. Ouyang, T. et al. Heterostructures composed of N-doped carbon nanotubes encapsulating cobalt and β-Mo_2C nanoparticles as bifunctional electrodes for water splitting. *Angew. Chem. Int. Ed.* **58**, 4923-4928 (2019).
68. Lai, W. H. et al. General π-electron-assisted strategy for Ir, Pt, Ru, Pd, Fe, Ni single-atom electrocatalysts with bifunctional active sites for highly efficient water

splitting. *Angew. Chem. Int. Ed.* **58**, 11868-11873 (2019).
69. Yao, Q. et al. Channel-rich RuCu nanosheets for pH-universal overall water splitting electrocatalysis. *Angew. Chem. Int. Ed.* **58**, 13983-13988 (2019).
70. Menezes, P. W. et al. Structurally ordered intermetallic cobalt stannide nanocrystals for high-performance electrocatalytic overall water-splitting. *Angew. Chem. Int. Ed.* **57**, 15237-15242 (2018).
71. Zhao, X. et al. Bifunctional electrocatalysts for overall water splitting from an iron/nickel-based bimetallic metal-organic framework/dicyandiamide composite. *Angew. Chem. Int. Ed.* **57**, 8921-8926 (2018).
72. Wu, R. et al. A janus nickel cobalt phosphide catalyst for high-efficiency neutral-pH water splitting. *Angew. Chem. Int. Ed.* **57**, 15445-15449 (2018).
73. Ledendecker, M. et al.The synthesis of nanostructured Ni_5P_4 films and their use as a non-noble bifunctional electrocatalyst for full water splitting. *Angew. Chem. Int. Ed.* **54**, 12361-12365 (2015).
74. Li, M. X. et al. Ni strongly coupled with Mo_2C encapsulated in nitrogen-doped carbon nanofibers as robust bifunctional catalyst for overall water splitting. *Adv. Energy Mater.* **9,** 1803185 (2019).
75. Zhu, C. L. et al. Fe-Ni-Mo nitride porous nanotubes for full water splitting and zn-air batteries. *Adv. Energy Mater.* **8,** 1802327 (2018).
76. An, L. et al. Epitaxial heterogeneous interfaces on N-$NiMoO_4$-NiS_2 nanowires nanosheets to boost hydrogen and oxygen production for overall water splitting. *Adv. Funct. Mater.* **29,** 1805298 (2019).
77. Hou, J. et al. Promoting active sites in core–shell nanowire array as mott–schottky electrocatalysts for efficient and stable overall water splitting. *Adv. Funct. Mater.* **28,** 1704447 (2018).
78. Hou, J. et al. Electrial behavior and electron transfer modulation of nichkel-copper nanoalloys confined in nickel-copper nitrides nanowires encapsulated in nitrogen-doped carbon framework as robust bifunctional electrocatalyst for overall water splitting. *Adv. Funct. Mater.* **28,** 1803278 (2018).
79. Sivanantham, A. et al. Hierarchical $NiCo_2S_4$ nanowire arrays supported on Ni foam an efficient and durable bifunctional electrocatalyst for oxygen and hydrogen evolution reactions. *Adv. Funct. Mater.* **26,** 4661-4672 (2016).
80. Wang, Z. et al. Phosphorus-doped Co_3O_4 nanowire array a highly efficient bifunctional electrocatalyst for overall water splitting. *ACS Catal.* **8,** 2236-2241 (2018)
81. Yu, F. et al. High-performance bifunctional porous non-noble metal phosphide catalyst for overall water splitting. *Nat. Commun.* **9**, 2551 (2018).
82. Xiao, X. et al. In situ growth of Ru nanoparticles on $(fe,ni)(oh)_2$ to boost hydrogen evolution activity at high current density in alkaline media. *Small Methods* **4**, 1900796 (2020).
83. Yu, L. et al. Ternary $Ni_{2(1-x)}Mo_{2x}P$ nanowire arrays toward efficient and stable hydrogen evolution electrocatalysis under large-current-density. *Nano Energy* **53**, 492-500 (2018).
84. Liang, C. et al. Exceptional performance of hierarchical Ni–Fe oxyhydroxide@NiFe

alloy nanowire array electrocatalysts for large current density water splitting. *Energy Environ. Sci.* **13**, 86-95 (2020).

85. Jothi, V. R. et al. Corrosion and alloy engineering in rational design of high current density electrodes for efficient water splitting. *Adv. Energy Mater.* **10**, 1904020 (2020).
86. Senthil, Raja. D. et al. In situ grown bimetallic MOF-based composite as highly efficient bifunctional electrocatalyst for overall water splitting with ultrastability at high current densities. *Adv. Energy Mater.* **8**, 1801065 (2018).
87. Rossmeisl, J. et al. Electrolysis of water on (oxidized) metal surfaces. *Chem. Phys.* **319,** 178-184 (2005)
88. NIST-JANAF Thermochemical Tables. http://janaf.nist.gov/.
